# Detecting rare structural variation in evolving microbial populations from new sequence junctions using *breseq*

**DOI:** 10.3389/fgene.2014.00468

**Published:** 2015-01-21

**Authors:** Daniel E. Deatherage, Charles C. Traverse, Lindsey N. Wolf, Jeffrey E. Barrick

**Affiliations:** Department of Molecular Biosciences, Center for Systems and Synthetic Biology, Center for Computational Biology and Bioinformatics, Institute for Cellular and Molecular Biology, The University of Texas at AustinAustin, TX, USA

**Keywords:** genome resequencing, experimental evolution, insertion sequence, genetic parallelism, evolutionary dead end

## Abstract

New mutations leading to structural variation (SV) in genomes—in the form of mobile element insertions, large deletions, gene duplications, and other chromosomal rearrangements—can play a key role in microbial evolution. Yet, SV is considerably more difficult to predict from short-read genome resequencing data than single-nucleotide substitutions and indels (SN), so it is not yet routinely identified in studies that profile population-level genetic diversity over time in evolution experiments. We implemented an algorithm for detecting polymorphic SV as part of the *breseq* computational pipeline. This procedure examines split-read alignments, in which the two ends of a single sequencing read match disjoint locations in the reference genome, in order to detect structural variants and estimate their frequencies within a sample. We tested our algorithm using simulated *Escherichia coli* data and then applied it to 500- and 1000-generation population samples from the Lenski *E. coli* long-term evolution experiment (LTEE). Knowledge of genes that are targets of selection in the LTEE and mutations present in previously analyzed clonal isolates allowed us to evaluate the accuracy of our procedure. Overall, SV accounted for ~25% of the genetic diversity found in these samples. By profiling rare SV, we were able to identify many cases where alternative mutations in key genes transiently competed within a single population. We also found, unexpectedly, that mutations in two genes that rose to prominence at these early time points always went extinct in the long term. Because it is not limited by the base-calling error rate of the sequencing technology, our approach for identifying rare SV in whole-population samples may have a lower detection limit than similar predictions of SNs in these data sets. We anticipate that this functionality of *breseq* will be useful for providing a more complete picture of genome dynamics during evolution experiments with haploid microorganisms.

## Introduction

Mutations that cause large-scale structural changes in chromosomes may have very different roles in evolution than point mutations that alter only single DNA bases. Indeed, certain mutations creating structural variants (SVs) enable new adaptive phenotypes to arise in laboratory populations of microorganisms (Dunham et al., 2002; Gresham et al., 2008; Raeside et al., 2014), and some of these phenotypes may be completely inaccessible by point mutations alone (Blount et al., 2012). SV created by transposable elements has been shown to play a role in experiments examining bacterial adaptation to more complex environments such as the mouse gut (Barroso-Batista et al., 2014). Structural variants are also common during cancer progression (Yang et al., 2013) where they are known to be the driving mutations in cases such as the BCR-ABL gene fusion (Deininger et al., 2000). At the intersection of microbial and cancer evolution, SV created during the integration of human papillomavirus DNA into the human genome may contribute to oncogenesis (Woodman et al., 2007). In these cases, it may be important to profile newly evolved structural variation when it is still very rare within a cell population as a way of anticipating likely evolutionary outcomes in the future.

Laboratory experiments with microorganisms have proven to be powerful model systems for understanding evolutionary dynamics on a whole-genome scale using new high-throughput sequencing technologies (Conrad et al., 2011; Barrick and Lenski, 2013). Often, this analysis involves sequencing the genomes of individual strains isolated from evolving populations one at a time. Instead, DNA from entire populations of cells may be analyzed all at once to track genetic diversity. Recent surveys of this kind have revealed the complex evolutionary dynamics of new mutations involving genetic hitchhiking, clonal interference, fixation/extinction events, parallel evolution, and frequency-dependent interactions (Barrick and Lenski, 2009; Herron and Doebeli, 2013; Lang et al., 2013; Traverse et al., 2013). Whereas the computational techniques typically used to analyze short-read resequencing data in these studies are easily able to detect polymorphic single-nucleotide changes in genome sequences, structural variants have most often been analyzed in a more limited way by using approaches requiring prior knowledge of expected mutations, large reductions in read depth, or discovery by chance (Chubiz et al., 2012; Herron and Doebeli, 2013; Traverse et al., 2013). Unless SV was known and specifically targeted, these studies generally do not detect low and intermediate frequency SV. Therefore, they are likely missing a large pool of genetic diversity that could reveal information about both evolutionary dynamics and gene function.

Structural variation can theoretically be predicted from short-read genome resequencing data by examining several types of evidence (Deatherage and Barrick, 2014). Large deletions and duplications alter the level of read-depth coverage in specific regions of the reference genome. Duplications, deletions, inversions, translocations, and mobile element insertions can be detected by anomalous mapping of reads to the reference genome. When the boundary of the SV does not fall within a large repeat sequence, it may be possible to identify split-read alignments that extend across the junction, connecting distant regions of the reference genome (Ge et al., 2011; Kim and Salzberg, 2011; Kim et al., 2013). Alternatively, paired-end mapping may indicate that a sequenced DNA fragment has an unexpected insert size or orientation between the two terminal reads, which is also evidence that sequences in the sample have changed locations relative to the reference genome (Chen et al., 2009; Hormozdiari et al., 2010; Zeitouni et al., 2010). Finally, reads can be *de novo* assembled into contigs that are then compared to the reference genome to identify SV (Tenaillon et al., 2012).

Extending approaches that predict consensus SV in an individual haploid or diploid genome to predicting rare structural variation in a population sample that is a mixture of DNA from many individuals requires additional analysis. Tools such as TopHat2 exist that use RNAseq data to predict novel splice variants and estimate their frequencies within a sample according to a maximum likelihood algorithm that involves counting reads that overlap the new junction breakpoint (Kim et al., 2013). This is essentially the same computational problem as predicting SV representation in a haploid population, but this and most other tools for detecting SV are designed with eukaryotic genomes in mind. As a consequence, they make assumptions about the reference genome that make them difficult or impossible to use to predict polymorphic structural variation in microbial genomes. For example, they may specifically restrict their search space to identifying coding sequence gene fusions (Sboner et al., 2010; Ge et al., 2011) or report long lists of predictions with no information about what a reasonable score threshold is for accepting predictions for a particular sample (Kim and Salzberg, 2011). Even if these tools successfully predict newly juxtaposed regions of a bacterial genome, it is not always straightforward to manually decipher how these rearrangements correspond to a particular mutational event and annotate its effects on specific genes.

Here, we describe an algorithm that predicts polymorphic structural variation in population samples by examining split-read alignments. This analysis has been incorporated into the *breseq* computational pipeline for analyzing whole-genome DNA resequencing data derived from haploid microorganisms (Deatherage and Barrick, 2014). It extends previous work that developed a workflow and statistical model for identifying consensus mutations (those present at 100% frequency) in clonal samples (Barrick et al., 2014) to also predict and estimate the representation of structural variants present at low or intermediate frequencies in mixed-population samples. We validate the new algorithm on simulated *E. coli* resequencing data and further analyze its theoretical sensitivity for detecting rare variant sequence junctions. Then, we demonstrate the performance of this approach on sequencing data from the earliest archived time points of the 12 populations of the Lenski long-term evolution experiment with *E. coli* (LTEE) (Lenski et al., 1991; Lenski and Travisano, 1994). Overall, we identify 94 structural variants present in LTEE populations after 500 or 1000 generations of evolution. These SVs account for 28% of the mutations that were at a frequency of 5% or more within these populations at one of these two time points. By more completely profiling genetic diversity than has been possible in previous studies, we identify additional evolved alleles in many genes that were mutated in parallel across the 12 LTEE populations of this experiment. Interestingly, two genes with beneficial mutations that transiently rose to prominence early in the LTEE in multiple populations were never mutated in the *E. coli* lineages that were ultimately successful.

## Materials and methods

### Predicting variant junctions

Version 0.25 of the *breseq* computational pipeline was used in this study (Deatherage and Barrick, 2014). The source code is freely available online (http://barricklab.org/breseq). Candidates for new sequence junctions supporting structural variation were identified using an algorithm that is fully described elsewhere (Barrick et al., 2014). Briefly, DNA sequencing reads in FASTQ format are first mapped to the reference genome using Bowtie2 (Langmead and Salzberg, 2012) with parameters that report partial alignments and matches to repeat sequences. Then, split-read alignments, in which the two ends of a read map sufficiently well to disjoint reference locations, are used to construct a list of potential variant junctions that may be present in the sample. All input reads are then re-mapped to determine whether they are “spanning reads” that support the existence of a new variant junction because they align better to it than to anywhere in the reference genome.

**Figure 1:**
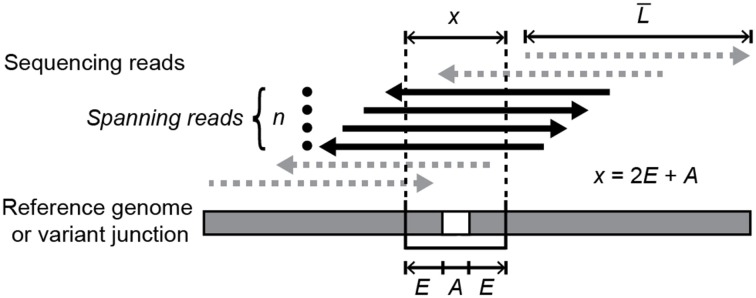
**Requirements for spanning reads that support a variant junction sequence**. In order for a mapped read to count as evidence—either for a new variant junction created by a structural mutation or for a mutually exclusive sequence that supports the unmutated reference genome—it must cross the junction breakpoint and continue to perfectly align for least a specified extension length (*E*) on each side of the junction past any ambiguous bases (*A*) at the breakpoint. Ambiguity occurs when reads with alignments ending in this region match the reference genome and putative variant junction equally well. When estimating the representation of a new junction sequence in a sample that is a mixture of reference and variant genomes, the counts of spanning reads for each junction (*n*) must be normalized by the relative chances of observing spanning reads for that junction. The target size used in this normalization is the average read length in the input data set (*L*) minus the total length a read must extend through to meet the spanning requirement (*x*) plus one.

In the default “consensus” mode described previously, *breseq* assumes that new genetic variants are present at a frequency of 100% in the sequenced DNA sample because it is clonal. Thus, it rejects candidate junctions for which the set of all spanning reads appears skewed or insufficient relative to a typical position in the reference genome (Barrick et al., 2014). When the new “polymorphism” mode described here for junctions is activated, *breseq* requires only that there be a number of spanning reads (*n*) that meets or exceeds a minimum threshold (default: 3) to predict a polymorphic junction (Figure 1). As in consensus mode, a spanning read must match the new junction sequence better than the reference genome sequence by aligning past the breakpoint by a number of bases greater than the number of ambiguous bases (*A*), which match either the reference sequence or the putative variant junction equally well. However, to qualify as a spanning read in polymorphism mode, a read must also continue to perfectly match for a certain number of additional “extension” bases (*E*) past this point (default: 6). These settings yielded high quality junction predictions in this study, as detailed in the results section. The *n* and *E* parameters can be set by the user as command-line options to potentially improve performance on other data sets.

### Estimating the frequencies of structural variants

For each predicted junction, *breseq* estimates its frequency within the genomes present in a population sample by comparing the number of reads spanning the new junction to the number of reads spanning each of the locations brought together by the new junction in its original context in the reference genome. Normalized read counts (*f*) are first calculated for each variant (*f*_V_) and reference location (*f*_R_) associated with a junction by dividing by the relevant “target size,” the number of possible positions where a read with the average length (*L*) in the input data set could start and qualify as a spanning read:

(1) $$f=\frac{n}{\bar{L}-(2E+A)+1}$$

The frequency of each new junction is then estimated as the normalized read count for the new variant junction divided by the average of the normalized read counts for the two reference locations. If we define *N*_V_ as the number of variant junctions and *N*_R_ as the number of reference locations matching sides of the new junction that are informative for calculating its frequency, *N*_V_ = 1 and *N*_R_ = 2 in this most basic case. However, when one side of a new junction matches a repeat region (e.g., a bacterial insertion sequence) in the reference genome, this side of the junction is uninformative for estimating its frequency and is omitted from the calculation (leaving *N*_R_ = 1). We further define *f*_V,i_ and f_*R*,*i*_ as the normalized read counts for the *i*-th informative variant junction and reference locations, respectively. Thus, the general equation for the frequency of a junction is:

(2) $$F_{\text{v}}=\frac{\frac{1}{N_{\text{V}}}\sum_{i\;=\;1}^{N_{\text{V}}}f_{\text{V},\text{i}}}{\frac{1}{N_{\text{R}}}\sum_{i=1}^{N_{\text{R}}}f_{\text{R},\text{i}}+\frac{1}{N_{\text{V}}}\sum_{i\;=\;1}^{N_{\text{V}}}f_{\text{V},\text{i}}}$$

**Figure 2:**
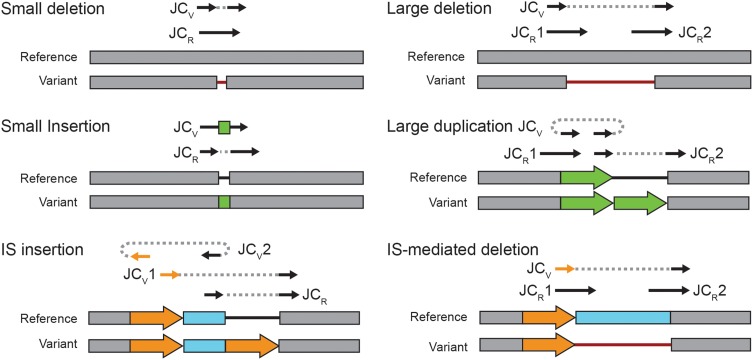
**Estimating structural variant frequencies in population samples**. Each panel shows a type of structural mutation that can be predicted from new sequence junctions. The reference and variant lines show the configurations of the two alternative genomic states. Reads supporting all possible mutually exclusive junctions present in either the reference (JC_R_) or the variant (JC_V_) are depicted schematically above these two states. Solid lines with arrows show how a read would align to the reference genome for each of these junction possibilities. Two sides of the same read are connected with a dashed line when they map to discontinuous locations. In the genome tracks, block arrows of the same color are repeats of the exact same sequence. Thin lines are gaps in the respective sequence to account for insertions and deletions. To estimate the frequency at which a structural variant is present in a population sample, normalized counts of spanning reads for each relevant JC_R_ and JC_V_ are combined using Equation 2.

New junction (JC) evidence can be used to predict several types of structural variation (Figure 2). By default, *breseq* is conservative in predicting genetic diversity within a population from JC evidence. In polymorphism mode, it will only use junctions to predict new mobile element insertions and small duplications, insertions, or deletions with sizes that are less than the average read length. The one exception is that a junction will be used to predict a larger consensus (100% frequency) deletion when the region that it spans in the reference genome lacks read coverage, i.e., produces missing coverage (MC) evidence (Barrick et al., 2014). All other JC evidence items are reported as “unassigned” to any mutation in the *breseq* results for the user to manually evaluate.

Insertions of new copies of mobile elements—most commonly insertion sequence (IS) elements in bacteria—are predicted from two JC items. One side of each JC must be located within 10 bases of the other in the reference genome in the proper orientation and the other side of each must map to the opposite boundary of the same mobile element family. Because two JC predictions are required to predict a mobile element insertion, the normalized spanning read counts for each new junction and its corresponding original reference sequence alternatives are pooled (such that *N*_V_ = 2 and *N*_R_ = 2) to calculate the overall frequency of this type of SV within a population from Equation 2.

For the purpose of identifying SV in the study of the LTEE, we further promoted unassigned JC evidence to predictions of likely mutations as follows. In many cases only a single JC evidence was found mapping to the end of an IS boundary. This observation could be explained by two mutually exclusive situations. First, it might indicate simply that one of the two real junctions was not recovered by chance, because it was present at a low frequency near the stochastic sampling limit. Second, if this junction is associated with an IS-mediated deletion extending from one end of an existing IS element (Figure 2), then there would only be one *bona fide* variant junction. In this case, an examination of the coverage of read depth might help to establish that there was actually a deletion for high-frequency variants, but the change in coverage depth will be imperceptible for rare variants. Therefore, we classified most singleton IS-related JC evidence items indeterminately, as either evidence for a new IS insertion or for an IS-mediated deletion mutation with the frequency assigned to the one JC evidence item. The one exception to this rule was that singleton JC evidence items were manually converted to deletion mutations in the ribose utilization operon, as an upstream IS*150* element is known to mediate very common deletions in this region (Cooper et al., 2001).

A few junctions were manually excluded from the analysis for technical reasons. Most appeared to be derived from insertions of one IS element into another IS element. Since both sides of these JC are in repeat regions, the frequencies of these putative structural variants cannot be estimated from spanning reads. Additionally, one junction was predicted with the two sides of the junction matching within the *yehV* gene in an outward orientation that is not compatible with any of the canonical types of mutations. We have seen similar, apparently spurious predictions in other data sets. As the two sides of the junction are always within a distance that is roughly the same as the sequenced DNA fragment size, we believe that the reads supporting these JC predictions may be related to some sort of rare but systematic PCR amplification or ligation artifact during Illumina library preparation.

The IS-mediated ribose operon deletions observed in the LTEE population data sets also illustrate a special case of nested deletions, in which the frequencies of variants predicted from new junctions need further correction. Each of these deletions extends from the end of the same IS*150* element to a different downstream location. When two different deletions occur in distinct subpopulations within the same bacterial population, the calculated frequency of the outer deletion (with an endpoint farther from the IS*150* element) is accurate, but the frequency of the inner deletion is overestimated because it is actually only present at this frequency within the subpopulation that does not have the outermost deletion. Therefore, the frequencies of nested ribose deletions were corrected by multiplying them by one minus the sum of the frequencies of all deletions that completely contained them. Examining the read-depth of sequencing coverage across these regions agreed with these ribose deletion frequency estimates. No other nested deletions appear to exist within this data set.

### Simulated data sets

We simulated two types of model data sets from the *E. coli* B strain REL606 genome sequence (GenBank:NC_012967.1) (Jeong et al., 2009). Two separate reference sequences were used to simulate reads: (1) the unmutated REL606 genome and (2) an *in silico* mutated REL606 genome with 100 random deletions (REL606Δ), each one removing exactly 1,000 base pairs. These deletions were restricted to locations in the original REL606 genome that were outside exact repeats of 50 or more bases identified using MUMmer (version 3.23) (Kurtz et al., 2004). The *gdtools* command packaged with *breseq* was used to simulate these mutations.

For the first “tiling” read data set, we systematically created error-free 50-base reads beginning at every position, on each strand, for both REL606 and REL606Δ using a custom Python script. Thus, each of these tiled read files had exactly 100-fold overall sequencing coverage of the respective reference genome. Concatenating these two read files created a mock sample in which each of the 100 deletions in REL606Δ was present at a frequency of exactly 50%. This data set was used to test the algorithm in *breseq* for calculating new junction frequencies from spanning reads to be sure that it accounted properly for the spanning read requirements, including adjustments to the target size for possible spanning reads due to overlap between the two sides of the junction, as described above.

For the second “sensitivity” data sets designed to test the detection of low frequency structural variants by *breseq*, we used the Mason read simulator (version 0.1.2) in Illumina mode (Holtgrewe, 2010). We created two types of test data sets: one with completely error-free reads and one with more realistic reads that were generated with simulated errors, including base mismatches and indels, according to the default settings for Mason. Again, we generated reads separately for the REL606 and REL606Δ reference sequences and combined them to simulate population samples. To obtain 100-fold total read depth, we generated reads from REL606Δ at 1-, 2-, 4-, 8-, 16-, and 32-fold coverage, which were then combined with reads from REL606 at 99-, 98-, 96-, 92-, 84-, and 68-fold coverage, respectively. Simulated read files for each combination of the two error models and six variant genome coverage depths were generated in triplicate.

### Theoretical chances of detecting structural variants

We performed a statistical analysis to establish the theoretical limitations of the algorithm implemented in *breseq* for detecting rare structural variants. There are three input parameters for this model. The first is the average read length (*L*). The second is the average read-depth coverage for the genomic variant containing a new junction (*C*). The third is a size parameter (*r*) for a negative negative binomial fit to the observed distribution of read-depth coverage at different positions in the reference genome. The *r* parameter accounts for biases in the coverage of reads sampled from different regions of a genome. For example, PCR amplification biases during Illumina library preparation are known to lead to systematic undersampling of sequencing reads derived from DNA fragments with extreme GC-content (<10 or >60%) (Aird et al., 2011). Smaller values of *r* indicate greater overdispersion (variance) relative to the ideal case of a Poisson distribution in which reads are sampled uniformly across the reference genome (for which the *r* parameter is infinite).

In order for *breseq* to predict a variant sequence junction, there must be at least two reads with split-read alignments across the breakpoint so that it is initially identified as a candidate (Barrick et al., 2014). With the Bowtie2 mapping parameters used by *breseq*, the minimum length of each of these two matches to the reference genome is 6 + 0.2 *L* bases. Therefore, for any given read overlapping a position near the junction breakpoint in the variant genome, the probability that it extends far enough into each side of the junction to map twice is 0.6–11/*L*. To meet the requirement for predicting a polymorphic junction, at least one additional read (for three total) must map to the reconstructed candidate junction sequence during the second alignment phase and qualify as a spanning read. If we discount the usually negligible size of any stretch of ambiguous bases at the new junction breakpoint (*A*) for this calculation, the probability a mapped read will count as a spanning read is 1–(2*E*–1)/*L*. For the default value of *E*, this probability is higher than the chance of a read mapping to both sides of the junction during the initial step. Note that an additional assumption in all of these calculations is that sequencing errors have little effect on correctly mapping and aligning most reads in a data set. Neglecting this added complexity for Illumina data, at least, is justified because there is typically only one base error in every few hundred sequenced bases, and many such errors would not prevent a read from mapping to both sides of or spanning a junction.

To predict the theoretical sensitivity of *breseq* on sequencing data with different characteristics (in terms of *L*, *C*, and *r*), we summed the joint probabilities of observing a given variant genome coverage value for the new junction of interest from the relevant negative binomial distribution and the chances that there would be at least two twice-mapped reads and at least one more twice-mapped or spanning read at that level of coverage. These latter probabilities were calculated from binomial distributions with the chances that an individual read overlapping a position at the junction breakpoint would be twice-mapped or spanning determined as explained above. In the context of analyzing the 100-deletion simulation data set, we also used this model with a further step that converted the expected sensitivity values into a 95% confidence band of exact binomial confidence limits using each read-depth specific model probability and 100 total trials. All statistical calculations were performed using R version 3.1.1 (R Core Team, 2014).

### LTEE population sequencing

Genomic DNA was isolated from 500- and 1000-generation LTEE population samples archived in 15–20% (v/v) glycerol. A volume of each sample sufficient to provide a fully representative cell population (120 μl)—as would be transferred daily during the LTEE—was washed to remove residual glycerol by pelleting cells by centrifugation, removing the supernatant, and washing once with Davis Minimal (DM) medium (Lenski and Travisano, 1994). The resulting cell pellet was resuspended in 10 ml of DM supplemented with 1 g/L glucose (DM1000) and grown overnight at 37°C with orbital shaking. Genomic DNA was isolated from the resulting cultures using a PureLink Genomic DNA Mini Kit (Life Technologies) and fragmented using a Covaris S2 focused-ultrasonicator. DNA samples were prepared for paired-end sequencing using the NEBNext genomic library prep kit for Illumina (New England Biolabs). Sequencing on an Illumina HiSeq 2000 instrument at the University of Texas at Austin Genome Sequencing and Analysis Facility generated 2 × 101-base paired-end reads for each sample with an average insert size of 180–260 bases. Raw sequencing reads have been deposited in the NCBI Sequence Read Archive (Accession SRP047501).

To predict genetic variation in these samples, Illumina adaptor sequences were trimmed from the ends of reads using Flexbar (Dodt et al., 2012). Then, individual bases with quality scores of <20 or 10-base windows in which the average base quality score was <20 were trimmed from the ends of reads using Trimmomatic (version 0.32) (Bolger et al., 2014). The resulting final trimmed reads had an average length of ~80 bases for all 24 datasets. These reads were analyzed by *breseq* in polymorphism mode with respect to the REL606 genome sequence. REL606 was the ancestral strain used to initiate half of the LTEE populations. The other half of the populations were initiated from strain REL607, which differs by two point mutations (in *araA* and *recD*) from REL606. These two genetic differences were detected as consensus mutations in all REL607-derived populations, and were not included in the analysis of *de novo* mutations that occurred during the LTEE.

Polymorphic structural mutations were predicted in these samples as described above. Single-nucleotide substitutions and single-base insertion or deletion (indel) mutations were predicted by *breseq* using previously described methods (Barrick and Lenski, 2009). Briefly, re-calibrated error rates estimated from each dataset were used in a Bayesian SNP caller that includes states for indels. Mutations passing an expectation score cutoff were further filtered using a Kolmogorov-Smirnov test for bias in the qualities of bases supporting the new variant and Fisher's exact test to check for bias in the strands of reads supporting the new variant, both rejecting the mutation if it was biased at a *p* = 0.05 significance level. To only examine the highest-quality predictions we further restricted all of our analyses to mutations with a frequency of ≥5% within each sample, except in some of the analyses of genetic parallelism where we included lower-frequency structural variant predictions. We also disregarded indel mutations that extended or contracted a homopolymer repeat of three or more bases in the reference and removed single-base changes when they created a homopolymer repeat of five or more bases with at least one base in the repeat on each side of the mutated position. These discrepancies are likely due to sequencing errors and seemed to occur most commonly due to the ends of sequencing reads becoming homopolymers in regions that mapped to near-homopolymers.

### LTEE isolate *rbs* genotyping

Frozen whole-population samples archived after 500 or 1000 generations of the LTEE were spread onto DM-glucose agar and incubated overnight at 37°C. From each sample, colonies were randomly picked and genotyped for ribose deletions by whole-cell PCR. Two different PCR reactions were conducted for each isolate. The first reaction used primers #1 (5′-TGCCGGATGATGGAAACCTC) and #2 (5′-AACCAGTTTCAGATCAACCGG) to amplify a DNA fragment that is 5,603-bp long in the ancestral strain. The second used primers #1 and #3 (5′-GATGGCCTTCTTCATGCAGG) to amplify an overlapping DNA fragment that is 9,010-bp long in the ancestral strain. Small deletions were assigned using size changes in the first PCR product, whereas larger deletions were genotyped as changes in the size of the second PCR product. In total, we genotyped 18 and 41 clones from Ara-3 at 500 and 1000 generations, respectively, and 73 and 77 clones from Ara-5 at 500 and 1000 generations, respectively. Representative isolates with ribose operon deletions giving different changes in the sizes of PCR product bands were Sanger sequenced with primer #1 to determine the precise chromosomal bases deleted. Clones from Ara-1 isolated at 500 and 1000 generations were genotyped in a similar manner in a previous study (Woods et al., 2011).

### Fixed LTEE mutations

We used *breseq* in consensus mode with default parameters to predict mutations in published whole-genome resequencing data sets for 17 clonal isolates from 8 of the LTEE populations that had been used previously to analyze synonymous mutation rates (Wielgoss et al., 2011). Unassigned missing coverage evidence items and unassigned new junction evidence items were manually examined for each sample to produce a complete list of mutations, using approaches described in detail elsewhere (Deatherage and Barrick, 2014). All 17 of these clones were isolated at late time points, after 20,000–40,000 generations of evolution and before hypermutator phenotypes arose in the respective populations. Thus, most new mutations in these genomes are expected to be adaptive (Barrick et al., 2009). The clones sequenced from the same population at the same time point were nearly genetically identical in all cases. This observation and past studies of genetic diversity in the LTEE populations over time (Barrick and Lenski, 2009; Woods et al., 2011; Blount et al., 2012) support the assertion that mutations observed by 1000 generations that are still present after ≥20,000 generations are most likely fixed, i.e., present in 100% of the population at the later time point. The populations analyzed in this way did not include a known exception to this rule: population Ara-2 diverged into two clades that co-existed with one another for tens of thousands of generations (Rozen et al., 2005).

## Results

### Algorithm performance

As described in the Materials and Methods, we added functionality for detecting polymorphic variant junctions indicative of structural mutations in high-throughput genome resequencing data to the *breseq* computational pipeline (Barrick et al., 2014; Deatherage and Barrick, 2014). Our method uses reads that connect disjoint regions in the reference genome (Figure 1) to predict newly juxtaposed sequences that are evidence for various types of structural variation in the sample (Figure 2). It is important to be clear at the outset that this algorithm cannot detect certain instances of SV, such as inversions or deletions, if they involve recombination events between exact genomic repeats with lengths on the same order as the DNA sequencing reads that are being analyzed. We first evaluated the performance of this algorithm using simulated DNA sequencing data and theoretical calculations.

### Simulated data

We validated the implementation of our algorithm using two different types of simulated data sets. The first “tiling” data set consisted of a complete set of error-free 50-base reads beginning at every position and on each strand in both a reference *E. coli* genome (REL606) and a variant genome in which 100 random 1000-bp deletions were computationally introduced (REL606Δ). Running *breseq* on this data recovered all 100 polymorphic variant junctions in REL606Δ with no false-positive predictions of other junctions. Importantly, each variant junction was also correctly predicted to be present at the designed frequency of exactly 50% within the sample. This result demonstrates that corrections for different target sizes of spanning reads that can support reference vs. variant junctions, due to ambiguity at breakpoint sequences, are properly calculated for a variety of test cases.

The second “sensitivity” data sets consisted of mock Illumina reads generated by the Mason read simulator (Holtgrewe, 2010). In contrast to the perfectly even coverage of the “tiling” data set, these reads are sampled randomly from different locations across the reference genome sequence. To examine how this unevenness combined with a much more limited representation of the variant genome in a sample affected *breseq*'s ability to detect the same 1000-bp deletions in the REL606Δ genome, we simulated read data sets with a range of average read-depth coverage of the variant genome from 1 to 32 in triplicate. In each case, we added reads generated from the unmutated reference genome to achieve a total coverage of 100-fold, but it is important to note that including any number of additional reference reads is not expected to affect the sensitivity of our algorithm, i.e., its ability to recover rare variant junctions (the number of false-negative predictions). A variant coverage of 1.0 applies to a junction present at a frequency of 1% in a data set with a total of 100-fold average read-depth coverage. It also applies equally well to a variant frequency of 0.1% in a data set with 1000-fold average coverage, 0.01% frequency with 10,000-fold coverage, and so on.

**Figure 3:**
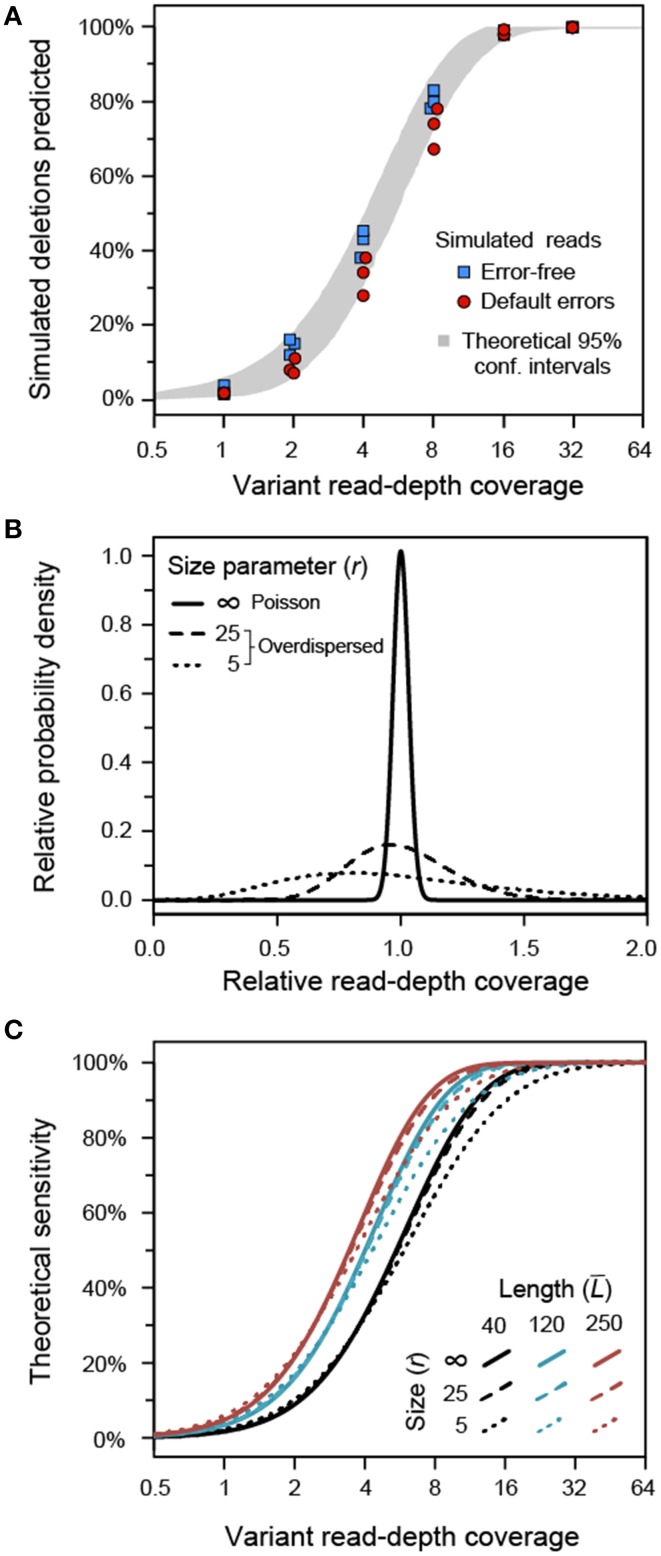
**Sensitivity of variant junction detection. (A)** Illumina read data sets with 100-fold read-depth coverage were simulated for population samples consisting of a mixture of an *E. coli* reference genome and a variant genome with 100 random deletions. The average read-depth of the variant genome within the sample determined how many of these deletions were detected. In data sets in which no simulated sequencing errors were present in the reads (blue squares), the number of deletions detected matches theoretical calculations based on the requirement that there be at least three spanning reads and the random distribution of the simulated reads across the genome (95% confidence intervals in gray). When typical Illumina sequencing errors were simulated (red circles), the sensitivity was slightly decreased, presumably because some errors prevent a read from mapping or aligning adequately to be counted as support for a new prediction of a variant junction. The precision of the algorithm on all of these model datasets was 100% (there were no false-positive predictions). **(B)** Distributions of variant genome coverage that would be observed at different positions in a genome given an average value of one for different values of the negative binomial size parameter (*r*) representing overdispersion due to biases in coverage at different sites. An idealized Poisson distribution representing no bias is shown at *r* = ∞. **(C)** Theoretical sensitivity of the algorithm employed by *breseq* for detecting variant junctions as a function of read length (line color) and negative binomial distribution size parameter for coverage bias (line dash style). These calculations assume completely error-free reads.

When the input reads were simulated with no sequencing errors, the sensitivity of *breseq* agreed well with theoretical calculations of the algorithm's performance assuming a random (Poisson) distribution of read-depth coverage across different positions in the reference genome (Figure 3A), as described in the next section. Data sets generated in the same way, but with default Illumina reads as simulated by Mason—which include base mismatch, insertion, and deletion errors—had significantly reduced sensitivity across all variant genome coverage levels (one-tailed Wilcoxon signed rank test, *p* = 0.0012), although the effect is not great (Figure 3A). For example, at a 4-fold variant genome coverage depth the error-free reads detected an average of 42% of the new junctions created by the simulated deletions. It was still possible to detect an average of 33% of these variant junctions from the reads containing errors. In both cases, ≥16-fold variant read depth recovered nearly all of the variant junctions. For all of the simulated Illumina read data sets, there were no false-positive predictions of junctions that did not exist in the variant genome. However, if there were a much higher level of read-depth coverage than the 100-fold total present in these data sets, it is possible that sequencing errors could lead to mismapping of enough reads that spurious junctions would be predicted, decreasing the precision of our algorithm as discussed further below.

### Theoretical analysis of sensitivity

To further analyze the limits on the sensitivity of our algorithm, we derived a formula for calculating the theoretical chances that any given junction would be predicted from the reads present in a particular sequencing data set (Materials and Methods). This model was used to understand how the detection of rare structural variants would be affected by three factors: (1) read length, (2) average coverage of the variant genome within the sample, and (3) skewed over- and under-representation of reads derived from different genomic locations. The final issue of non-uniformity in how reads are sampled from a genome leads to increased variance in read-depth coverage levels across a genome in real data sets relative to an idealized Poisson distribution in which reads are uniformly sampled (as was the case for the mock data sets created using the Mason read simulator). This skew can, for example, be related to PCR biases during library preparation (Aird et al., 2011). The resulting distribution of coverage can be fit using a negative binomial distribution that includes a size parameter (*r*) that is smaller the more overdispersed the distribution is relative to an idealized Poisson distribution with no bias (*r* = ∞) (Barrick and Lenski, 2009). Examples of coverage distributions, including a Poisson distribution and two with size parameters more typical of the experimental data sets analyzed in the following section, are shown in Figure 3B.

All else being equal, longer reads are more likely to successfully detect a candidate junction than shorter reads because of the requirements for at least two reads that map disjointly across the junction boundary and at least three reads that span at least six bases past the breakpoint. However, we found that increasing the size of the reads from 40 to 250 base pairs in our model only slightly decreased the variant genome coverage needed to have a 50% chance of detecting a new junction: from 5.6 to 3.5 (Figure 3C). Skewed coverage depth across the genome does degrade the ability to detect some rare variants, as expected, but this effect is even more subtle. Biased coverage prevents recovering the last few remaining unpredicted variants when an overall high level of sensitivity has already been achieved. For example, with 40-base reads 16.3-fold average coverage of the variant genome is required to have a 90% chance of recovering a variant junction in a high bias data set (*r* = 5). The average variant genome read-depth required to achieve the same sensitivity is only reduced to 12.0 in a data set with no coverage bias (*r* = ∞).

Summing up the results of this analysis and the tests of simulated data: having longer reads, less-skewed coverage, or fewer sequencing errors in a genome resequencing data set is expected to have relatively little effect on the sensitivity of our algorithm for detecting new junctions indicative of structural variation, at least within the ranges tested. The primary consideration for detecting rare junctions in a sample is achieving a greater overall read-depth coverage of the genome.

### Analysis of LTEE populations

To more realistically evaluate the performance of our polymorphic structural variation prediction algorithm, we used *breseq* to analyze whole-population sequencing data from the Lenski long-term *E. coli* evolution experiment (LTEE). All 12 LTEE populations were analyzed at the two earliest time points at which samples were archived: after 500 and 1000 generations of evolution. An advantage of analyzing this data is that many whole-genome sequences of clonal isolates from these populations (Barrick et al., 2009; Blount et al., 2012; Wielgoss et al., 2013) as well as studies of genetic parallelism at specific loci (Cooper et al., 2001; Woods et al., 2006) are available for comparison. Large-scale structural variation has also been profiled in these populations using approaches that are based on technologies other than DNA sequencing (Raeside et al., 2014). Finally, the dynamics of specific mutations early in one LTEE population have been profiled in detail (Woods et al., 2011).

### Data set characteristics

**Figure 4:**
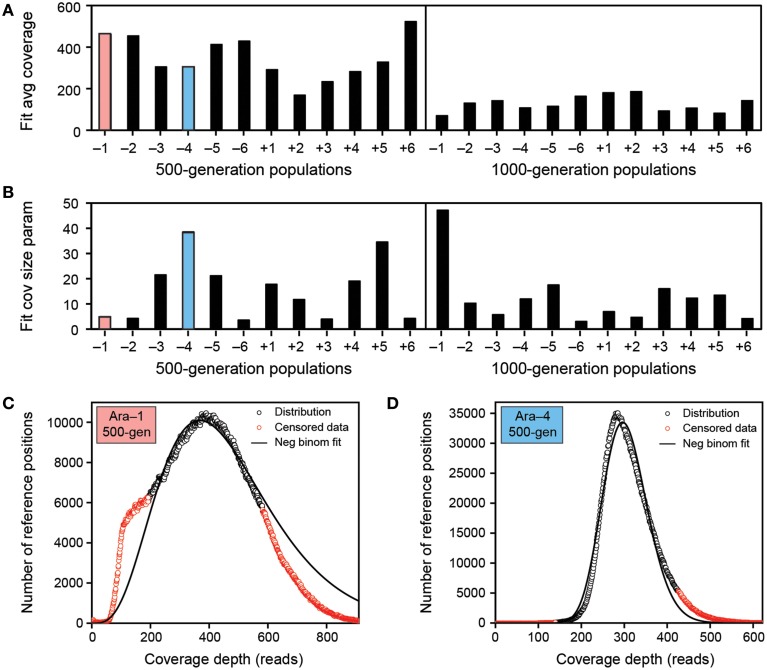
**Characteristics of the LTEE population sequencing data sets. (A)** Negative binomial fits to the distributions of read-depth coverage across the ancestral reference genome show that the average coverage was higher by a factor of approximately three in the 500-generations samples compared to the 1000-generation samples. **(B)** The uniformity of read-depth coverage across the reference genome varied greatly across these samples. This characteristic of the negative binomial distribution is captured by the size parameter (r) where lower values mean greater overdispersion relative to an idealized Poisson distribution in which there is an equal probability of reads being represented from all parts of the reference genome. **(C)** Negative binomial fit to the coverage distribution of population Ara-1 at 500 generations. This data set has a relatively large variance (small *r* size parameter of ~5), and it also has a distinct shoulder of many positions with lower-than-expected coverage values that is not fit well by this model. As described elsewhere, red values are censored during the fitting procedure to reduce the effect of potential outliers (Barrick et al., 2014). **(D)** Negative binomial fit to the coverage distribution of population Ara-4 at 500 generations. This data set has a relatively small variance (larger *r* size parameter of ~40) and yields a much better fit to the coverage read-depth model.

The Illumina HiSeq data sets for the 500-generation LTEE samples all had approximately 320-fold average read-depth coverage of the reference genome, while the 1000-generation samples all had only approximately 120-fold coverage (Figure 4A). There was much more variation between samples in how uniformly reads mapped across different regions of the reference genome, as characterized by the size parameter of negative binomial fits to each empirical distribution (Figure 4B). Some samples had a coverage distribution that was highly skewed (*r* ~ 5) (Figure 4C). Typically, these samples also exhibited a noticeable shoulder containing a significant number of genomic positions with lower-than-expected coverage relative to the fit. Other samples exhibited less-biased (narrower) coverage distributions that were more closely reproduced by the negative binomial fits (*r* ~ 25) (Figure 4D).

Given that the average read length in all LTEE samples was about 80 bases after preliminary adaptor and base-quality trimming steps, these coverage distribution characteristics can be used to calibrate, at least roughly, our expectations for recovering rare variant junctions in these samples by referring back to the theoretical treatment (Figure 3C). Thus, for a typical 500-generation sample (with size parameter *r* = 25), we predict that 95% of the variant junctions with frequencies >3.5% would be detected in a sample. Similarly, 50% with frequencies >1.3% and 5% with frequencies >0.4% would be recovered. In a typical 1000-generation sample, the equivalent 95%, 50%, and 5% sensitivity thresholds are reduced to mutations present at frequencies of 9.4%, 3.4%, and 1.0% in a sequenced population, respectively. Overall, we expect to detect nearly all of the structural variants that create new sequence junctions in the LTEE samples when they are present in at least 5% of the cells in a population and to also detect a majority of SV present at levels as low as 1%.

### Mutation predictions

In analyzing the LTEE data for polymorphic mutations, we examined point mutations and small indels in addition to structural variation to get a more complete picture of evolutionary dynamics and genetic diversity in these populations. We restricted SN predictions (encompassing all single-nucleotide substitutions, insertions, and deletions) to those predicted to be present in ≥5% of the population to account for the higher error rates in predicting these types of polymorphisms (Barrick and Lenski, 2009; Lang et al., 2013; Deatherage and Barrick, 2014). SV with an estimated frequency of <5% was included if it met the required criteria to be predicted by the algorithm implemented in *breseq* (Materials and Methods). Because *breseq* exclusively predicts insertions and deletions of ≥2 bases from new junction evidence (rather than from examining read pileups to the reference genome), we included even these relatively small mutations in the structural variation category.

**Figure 5:**
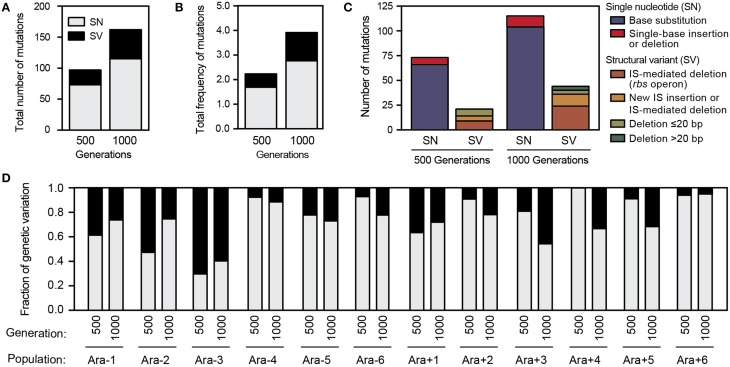
**Mutations predicted in LTEE 500- and 1000-generation populations. (A)** Total numbers of mutations detected across all 12 populations at each time point in each category. Single nucleotide (SN) mutations are single-base substitutions, insertions, and deletions. Structural variation (SV) mutations are insertions and deletions of ≥2 bases and other events such as IS element insertions, all of which are predicted from new sequence junctions by *breseq*. **(B)** Total frequencies of mutations detected across all 12 populations at each time point in each category. **(C)** Breakdown of the spectrum of molecular effects of all mutations detected within the 12 LTEE populations. **(D)** Fraction of total genetic variation contributed by SN vs. SV mutations in each LTEE population sample, weighted by mutation frequencies.

Overall, we detected a total of 142 SN mutations and 55 SV mutations that achieved a frequency of at least 5% in either the 500- or 1000-generation sample from each LTEE population. A total of 94 SV mutations were detected when including those below 5% frequency. Over the complete set of predicted polymorphisms, we rarely found the exact same genetic difference from the ancestor in multiple populations: there were 124 distinct SN alleles and 87 different SV alleles. We found slightly more SN and SV genetic variation in the 1000-generation samples compared to the 500-generation samples (Figure 5A), even though they had less sequencing coverage depth. Thus, genetic diversity appears to have generally been increasing in the LTEE through 1000 generations. When these mutations were weighted by their prevalence within each population and summed, there was almost no change in the relative representation of SN vs. SV mutations (Figure 5B). Therefore, there seems to be no gross disparity in which type of mutation was more likely to be successful during evolution, at least among the alleles that reached detectable frequencies. Extrapolating from these summed frequencies, a cell picked from one of the LTEE populations at 500 or 1000 generations would be expected to have ~2 or ~4 mutations, respectively.

In the aggregate, SV accounted for 28% of the genetic polymorphisms that reached a frequency of ≥5% within the LTEE populations at one of the time points that was profiled. We next examined in more detail what types of SV were predicted (Figure 5C). Roughly half of the detected SV was the result of IS-mediated deletions in the ribose utilization operon (*rbs*). Although *rbs* deletions confer only a small benefit relative to other beneficial alleles that are spreading through the LTEE populations at these early time points (Barrick et al., 2009; Khan et al., 2011), they occur at a very high rate by a mechanism involving an existing terminal IS*150* element and thus rapidly spread in all successful lineages in every LTEE population (Cooper et al., 2001). As expected, we often see different *rbs* deletions in competing lineages in the same LTEE population. The next most common category of SV involved other variant junctions compatible with IS-mediated deletions or new IS insertions (as diagramed in Figure 2). We also observed several non-IS-mediated deletions ranging in size from a few base pairs to 183 base pairs that created new variant junctions. The relative amount of SV within each population varied widely (Figure 5D). SV accounted for more than half of the genetic variation in population Ara-3 but was almost non-existent in populations Ara-4 and Ara + 6.

How accurate and sensitive are *breseq*'s predictions of structural variation? While the LTEE data sets are not a true “gold standard” in which the complete set of the polymorphic mutations that are present and their actual frequencies are known, comparing our predicted mutations with past studies does suggest that the SV prediction algorithm is performing well on these data sets. For example, it would be highly unlikely to predict so many variant junctions exactly joining the ends of IS-elements to other regions of the genome by chance (Figure 5C). In fact, the prevalence of these putative insertions of new copies of IS elements and IS-mediated deletions in the population samples is roughly the same as what has been seen in sequenced clonal isolates (Barrick et al., 2009). Over all 24 data sets analyzed, there was just one prediction in the raw SV output that was notably questionable. We removed this prediction before beginning our analysis for the reasons discussed below. More evidence related to evaluating the SV predictions is presented in the following sections.

**Figure 6:**
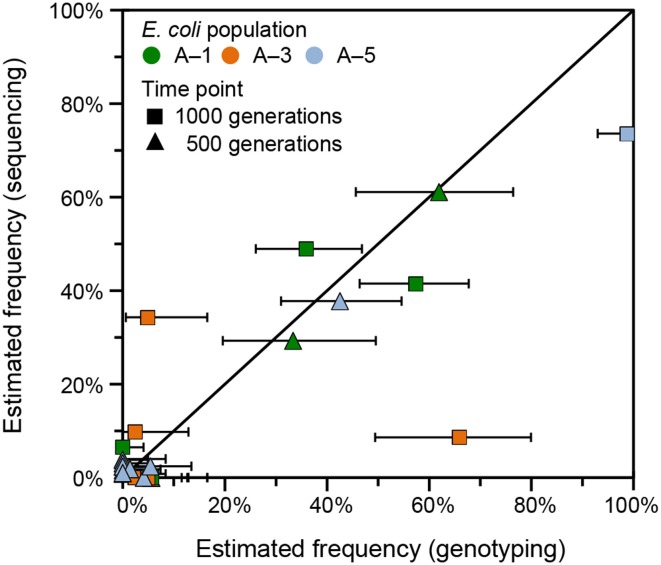
**Comparison of junction frequencies determined by genotyping and whole-population sequencing**. Deletions of various sizes extending from an IS*150* element into the adjacent ribose utilization operon are common in the LTEE. Each deletion generates a new variant junction. This plot compares the results of using polymorphic junction predictions in *breseq* and genotyping randomly picked clones to estimate the frequencies of the various deletion alleles of different sizes detected in three of the LTEE populations. Horizontal error bars are 95% exact binomial confidence intervals determined from the numbers of clonal isolates genotyped with and without a given ribose deletion allele. Estimation error in the vertical direction is likely of a similar magnitude, but it is difficult to estimate due to the nested nature of the ribose deletions (see Materials and Methods).

To examine the accuracy of the frequencies predicted from variant junctions for individual mutations in the LTEE populations, we compared the results from analyzing the high-throughput sequencing data with *breseq* to those from using PCR to genotype clonal isolates for *rbs* operon deletions. Many isolates from population Ara-1 had previously been tested for *rbs* deletions at 500 and 1000 generations (Woods et al., 2011). We genotyped additional clones isolated at both time points from populations Ara-3 and Ara-5 for this study. With these results, we have two independent estimates of the frequency of each *rbs* deletion allele. Note, however, that calculating the frequencies of *rbs* deletions (and determining confidence intervals for these estimates) from the variant junction analysis is complicated by how multiple deletions that are nested within one another are often present in the same population (Materials and Methods). Overall, there was reasonable agreement between the genotyping and sequencing estimates of allele frequencies for most *rbs* deletions (Figure 6). However, these estimates did appear to significantly disagree for at least three *rbs* deletions found in 1000-generation samples. It is not possible to completely pin these discrepancies on the junction-analysis algorithm, as there are also potential sources of error in the genotyping procedure. In particular, evolved LTEE clones with certain mutations may not survive plating on agar to isolate colonies or may more often give unscorable or ambiguous PCR genotyping results.

### Genetic parallelism

Even discounting *rbs* deletions, many of the polymorphic SV mutations predicted in the LTEE samples are concentrated in a relatively small number of *E. coli* genes (Table 1). This type of genetic parallelism is strong evidence that these mutations are adaptive (Tenaillon et al., 2012; Barrick and Lenski, 2013). As further evidence that the SV predictions are accurate, many of the genes with multiple SV polymorphisms are also commonly targets for SN mutations (e.g., *pykF*), despite the fact that SN and SV mutations are predicted by different methods. Finally, we tested whether these early polymorphisms were found in genes that were also mutated in clones isolated much later (after 20,000 generations or more) from the LTEE populations that had been sequenced individually. In general, the same genes had usually sustained mutations in these isolates. This result indicates that the types of beneficial mutations that initially rose to prominence were also successful in the longer run and eventually fixed within ~100% of the surviving cells in these populations. Another type of observation related to parallelism is illustrated by mutations in *nadR*. Polymorphic mutations in *nadR* were found in only four of the twelve 500- and 1000-generation LTEE samples, but the *nadR* gene was mutated in every later isolate tested. This result likely indicates that the first beneficial *nadR* mutations were just beginning to take over and reach detectable frequencies by 1000 generations.

**Table 1:** **Parallelism between early polymorphisms and fixed mutations in the LTEE**.

**Genes**	**Early polymorphisms**				**Fixed mutations**
	**Max freq (%)**	**SN alleles**	**SV alleles**	**Populations (%)**	**Populations**
*pykF*	>95	17	6	100	100%^*^
*rbsA–yieO*	>95	0	42	83	100%^*^
*spoT*	>95	9	0	67	64%^*^
*fabR*	>95	7	1	58	0%
*mraZ*	>95	2	5	58	0%
*mokB*	10	0	5	42	50%
*topA*	91	7	0	33	38%
*mreB*	85	5	0	33	38%
*nadR*	88	3	1	33	100%^*^
*ldrC*	6	0	3	25	63%
*mreC*	86	3	0	25	13%

Genes in which mutations were detected in least three LTEE populations in either the 500- or 1000-generation samples are shown. The numbers of different single-nucleotide substitution, insertion, or deletion alleles (SN) and structural variant alleles (SV) are totals across all 12 LTEE populations and both time points. The percentages of populations with fixed mutations affecting each of these genes were determined by analyzing the genomes of non-mutator clones isolated at ≥20,000 generations from eight LTEE populations (Wielgoss et al., 2011), except for cases marked by an asterisk (

* ) for which data from all 12 populations were summarized in a previous study (Barrick et al., 2009).

Early mutations in two genes—*fabR* (*yijC*) and *mraZ* (*yabB*)—exhibited the complete opposite trend from *nadR*. Mutations in these genes were common in the early LTEE samples: each occurred in seven of the 12 populations and reached near-fixation (>95% frequency) in one population. To achieve detectable frequencies so quickly and so many times in the LTEE, these mutations must have had highly beneficial effects on fitness in this environment. Despite this initial fitness benefit, cells with these alleles and any other mutations in these genes always went extinct by later in the evolution experiment. The dynamics of a key *fabR* allele were analyzed in one LTEE population in a previous study (Woods et al., 2011). In this case, a *fabR* mutation was found in genotypes that represented roughly half of the 500-generation Ara-1 population. By using evolutionary replay experiments, these *fabR*-containing genotypes were shown to be less able to improve in fitness after further evolution than competing subpopulations with alternative sets of beneficial mutations. The current results indicate that this phenomenon of *fabR* alleles creating “eventual loser” genotypes was apparently much more widespread in the LTEE than previously realized. Interestingly, our ability to detect polymorphic SV alleles revealed that mutations in a second gene (*mraZ*) also were highly successful early in the LTEE but were seemingly destined to be eventual losers in the long run.

**Figure 7:**
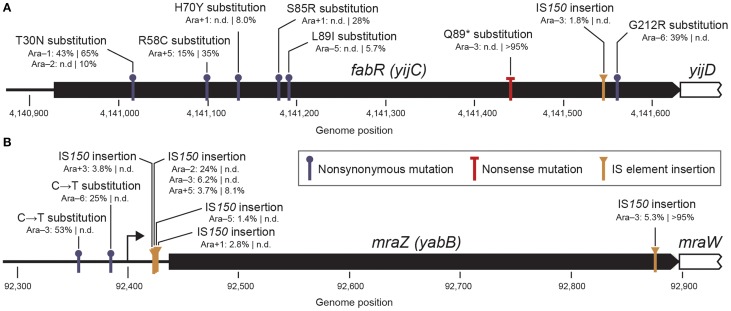
**Widespread early mutations that are ultimately unsuccessful. (A)** Details for mutations in the *fabR* (*yijC*) gene found in the LTEE. Following the Ara population designation, the estimated frequencies of each new mutation are shown at the two generational time points with a vertical bar between them (500|1000 generations). Some alleles were not detected (n.d.) at one time point. Mutations in this gene appear likely to generally lead to a loss of function, given that a high-frequency stop codon (Q89^*^) and an IS*150* insertion are observed within the protein reading frame. **(B)** Details for mutations affecting the *mraZ* (*yabB*) gene found in the LTEE. The *mraZ* transcription start site is shown as an arrow (Eraso et al., 2014). Mutations affecting the *mraZ* gene may be gain-of-function changes, as most are concentrated in the upstream region where they could alter transcriptional regulation.

Given the multiple different mutations observed in each of these target genes, we can predict what types of molecular effects they are having that are apparently highly beneficial to *E. coli* fitness when they initially occur but interact unfavorably with possible subsequent beneficial mutations. The previously detected *fabR* allele was a single amino acid substitution (Woods et al., 2011), which is relatively uninformative about its molecular effect. However, the full set of eight polymorphic *fabR* alleles detected in this study includes an IS*150* element insertion and a high-frequency mutation leading to a premature stop codon within the protein reading frame (Figure 7A). Therefore, the evolved *fabR* alleles appear to be loss-of-function mutations. FabR is a transcriptional repressor of fatty acid biosynthesis (Zhu et al., 2009). The reason that *fabR* mutations are beneficial for cellular fitness is not known.

In contrast to the case with *fabR*, most of the *mraZ* mutations are IS*150* insertions or single-base substitutions in the upstream promoter region (Figure 7B). Given the noticeable absence of point mutations in the reading frame, we hypothesize that these *mraZ* alleles do not simply reduce the activity of this gene. Even the one IS*150* insertion in the protein reading frame near the C-terminus, which reaches the highest frequency of any *mraZ* allele, may modulate rather than abolish activity. The *mraZ* gene encodes a transcriptional regulator that appears to affect cell wall biosynthesis and cell division processes (Eraso et al., 2014). Beneficial mutations affecting other genes involved in these processes—in the *mrdA* (*pbpA*) and *mrdB* (*rodA*) genes and the *glmUS* operon—have previously been characterized in LTEE strains (Philippe et al., 2009; Stanek et al., 2009). Other parallel mutations (*mreB* and *mreC*) affecting cell shape and size were also detected here as polymorphisms in the early LTEE populations (Table 1). Thus, it seems likely that the *mraZ* mutations have a beneficial effect related to the common physiological effects of all of these mutations on cell size and shape, but that the *mraZ* mutations have side-effects that preclude subsequent adaptive mutations in ways that mutations in those other genes do not.

### Mutational pathways and dynamics

**Figure 8:**
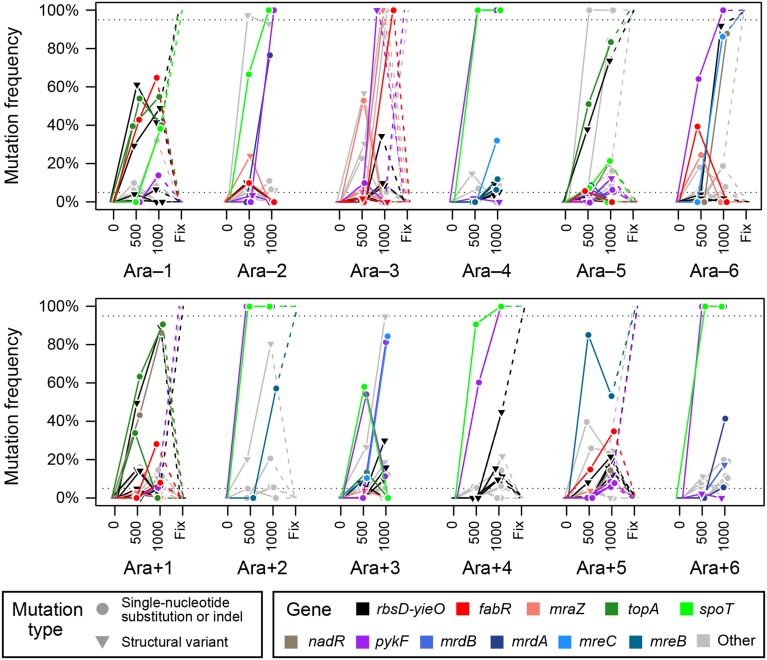
**Population-wide genetic diversity early in the LTEE**. Symbols indicate whether a mutation is a single-base substitution, insertion, or deletion (SN); or structural variant (SV). Colors indicate mutations in key genes, as discussed in the main text. The “Fix” column shows which alleles were present in clones sampled from these populations much later in the experiment (≥20,000 generations), presumably because they eventually swept to fixation and were present in 100% of the surviving population. Fixation data was only available for eight of the populations.

Tracking the early dynamics of all alleles in these populations reveals new information about how different combinations and orderings of mutations competed early in the LTEE (Figure 8). For eight of the populations, we were able to determine the long-term fates of these early alleles as extinction or fixation from whole-genome sequencing data from later clonal isolates (Materials and Methods). With respect to evaluating the performance of the SV prediction algorithm, we did not see any obvious cases of false-positive predictions that were incompatible with the expected asexual evolutionary dynamics. Mutations in diverged lineages are unable recombine into the same genetic background, so they should be consistent with perfectly nested sets of mutations. Thus, a false-positive prediction would be evident, for example, if one mutation were present at 50% frequency at both 500 and 1000 generations while a different mutation increased from 10% to 90% frequency. We also do not see any glaring omissions in the mutations predicted. For example, there are not any populations completely lacking high-frequency mutations early in the evolution experiment, which might have indicated that there were missing mutation predictions (i.e., false-negatives). Therefore, we believe that the polymorphic SV prediction procedure implemented in *breseq* accurately reveals all, or at least most, of the dynamics of important mutations in the LTEE population samples.

Across the 12 LTEE populations we observed several distinct patterns of mutational succession (Figure 8). The mutations that most commonly occurred together were *spoT* and *pykF*. In six populations, genotypes with these mutations were already a majority of the population by 500 generations, and in all but one of these cases (in Ara+3) their descendants have essentially completely taken over the population by 1000 generations. When the relative dynamics of these two mutations could be resolved, the *spoT* mutation appears to have occurred first, which would agree with a study of the *spoT* and *pykF* alleles that eventually reached fixation in population Ara-1 (Khan et al., 2011). It found epistatic interactions such that this particular *pykF* mutation was more beneficial in the context of other, earlier mutations including one in *spoT*. Another general pattern in these trajectories was that mutations affecting cell size and shape—in *mrdA*, *mrdB*, *mreB*, and *mreC* as discussed above—appeared to generally reach prominence during a second wave of beneficial mutations. Here, population Ara+5 was a notable exception in which an ultimately successful *mreB* mutation appeared to drive early evolution. Combinations of mutations including *pykF* and either *mreC* or *mreB* were successful in two additional populations (Ara-6 and Ara+5). Since these winning lineages did not include mutations in *spoT*, it is possible that there is some sort of functional interaction between this regulator and cell size and shape mutations (Philippe et al., 2007).

In at least three populations (Ara-1, Ara-3, and Ara+1) the most common mutations present at 1000 generations became extinct in the long run. In each of these cases and for other more minor subpopulations that reached a frequency of above 20% in four populations (Ara-2, Ara-3, Ara-6, and Ara+5), the loss of these genotypes seems to be explained by the presence of either a *fabR* or *mraZ* mutation. Note that this situation is likely true even in the case of Ara+1 where it is more difficult to recognize, but a *fabR* allele seems to be sweeping through the subpopulation that already has *topA*, *rbs*, and *nadR* mutations at 1000 generations. Future work will be needed to understand this striking result in which *fabR* or *mraZ* mutations are clearly very beneficial in the ancestral strain and in the context of certain other early beneficial mutations in the LTEE but appear to absolutely mark lineages as dead-ends that are destined for long-term extinction (Woods et al., 2011).

The Ara-3 LTEE population that eventually evolved citrate utilization (Blount et al., 2008) had the most interference from these eventual loser mutations. Here, there was first an unsuccessful *fabR*-containing clade that peaked near 500 generations and then a different lineage with both *fabR* and *mraZ* mutations that appeared to have fixed in the population by 1000 generations but was later replaced by descendants of cells with neither of these mutations that were apparently present at such a low frequency that they were not detected during this time (<1%). Since the Ara-3 population eventually fixed mutations in genes that are the typical LTEE targets of selection and citrate utilization evolved much later in the experiment after 30,000 generations (Blount et al., 2012; Quandt et al., 2013), it seems unlikely that these unusual early dynamics are related to why this population, alone out of all 12 LTEE populations, has achieved this rare metabolic innovation.

## Discussion

Testing of computational algorithms for predicting mutations from high-throughput sequencing data is generally hampered by the lack of experimental gold standard data sets. This is particularly true for understanding whether potential rare variants within a population are due to sequencing errors or are *bona fide* mutants. We used a combination of simulated high-throughput DNA sequencing data, theoretical calculations, and actual Illumina sequencing data for *E. coli* population samples from a long-term evolution experiment to evaluate an algorithm for predicting polymorphic structural variation. This analysis module is implemented as part of *breseq*, a computational pipeline with specialized functionality for examining resequencing data derived from haploid microbial genomes.

Predictions of rare single-base mutational variants in a population are fundamentally limited by the base error rate of the sequencing technology (Schmitt et al., 2012; Lou et al., 2013). In contrast, the prediction of structural variation from new-junction sequences should have a much lower error rate, as sequencing errors are highly unlikely to produce reads that map to discordant regions of a reference genome. In the current study, we observed no false-positive predictions on the simulated data and only one obvious false-positive prediction on the real *E. coli* data. In the future, it will be interesting to see how this algorithm scales to predictions of even rarer genetic variants that are potentially present at frequencies that are orders of magnitude below 1% of the population. On this scale, where genome-wide coverage depth can exceed 10,000-fold—which is easily achievable for viral genomes or targeted sequencing of specific genes or plasmids with current sequencing technologies—it is possible that new types of errors will begin to limit accurate SV detection.

From the current study, we anticipate at least two sources of errors that could result in unexpectedly high rates of false-positive predictions of new variant junctions in these more deeply sequenced data sets. First, when we did not stringently trim the 3′ ends of reads on base quality scores, there were sometimes enough spurious mismappings of reads to seed and support variant junctions that were clearly not biologically relevant. These spurious matches often involved homopolymer repeats or near-repeats at the ends of reads, which are commonly encountered errors in Illumina data sets. It may be desirable to categorically ignore these types of matches in future versions of *breseq* when seeding junctions if warranted by further analysis of the sensitivity-precision tradeoff. Second, much like PCR chimeras can arise that confound 16S rRNA sequencing (Schloss et al., 2011), it is possible that ligation and amplification steps in preparing a DNA sample for sequencing will introduce spurious junctions at some rate, and it is likely that these junctions will be biased such that they occur much more often in certain sequence contexts. How much these types of artifacts and others that we have not anticipated impact the effective detection limit of this algorithm on ultra-deep sequencing data sets remains to be seen.

There is also room for improvement in how SV allele frequencies are estimated by *breseq*. In particular, biased PCR amplification of inserts with extreme GC content will lead to inaccuracies in the estimated allele frequencies when the DNA fragments representing a variant junction vs. the reference junction have different base compositions. Horizontally transferred genes in particular are known to often have a significantly different GC content than vertically inherited chromosomal sequences (Daubin et al., 2003). Therefore, we expect that it will be especially difficult to accurately estimate the frequencies of junctions between these two types of heterologous regions within a given genome. It might be possible to correct somewhat for GC bias in future *breseq* versions by fitting an appropriate model to the genome-wide representation of DNA fragments with different properties. As importantly, *breseq* should output appropriate 95% confidence intervals on junction evidence and SV mutation frequencies to give the user an idea of the uncertainty in these estimates, particularly when the count of spanning reads supporting a structural variant is small.

The number of large-scale chromosomal changes that currently go undetected in studies of evolving populations of microorganisms that use whole-population sequencing to profile genetic diversity is largely unknown. Insofar as *E. coli* is a typical bacterium, our results predict that a sizeable proportion of the new mutations driving evolution may go undetected (~25%), although this number is expected to be highly variable, as it depends greatly on the number of active mobile genetic elements in a genome and the environment, among many other variables. By analyzing samples from the well-studied Lenski long-term evolution experiment with *E. coli* we have demonstrated how detecting SV can bolster analyses of genetic parallelism and provide information about how the activities of different genes are being altered by mutations to improve organismal fitness. Notably, more fully profiling SV diversity in a population can also reveal mutations in minor subpopulations that may be, for example, persistent evolutionary dead ends, sink populations that continually re-evolve from major ecotypes, or important but rare participants in ecological interactions that stabilize diversity. The improved functionality of *breseq* described here should help to close our current knowledge gap regarding SV in microbial evolution experiments.

### Conflict of interest statement

The authors declare that the research was conducted in the absence of any commercial or financial relationships that could be construed as a potential conflict of interest.

## Supplementary material

Data Sheet 1 **Genetic diversity predicted in the LTEE *E. coli* genomes**. Full details of all polymorphisms predicted by *breseq* are provided in one merged HTML comparison table and in individual files for each population sample in Genome Diff format. In the comparison table, the delta symbol (Δ) indicates that the mutation in the specified row is fully contained within a region that is deleted within the specified sample. The machine-parsable Genome Diff file format is fully described in the *breseq* documentation. Note that the *recD* and *araA* mutations present in the ancestors of all Ara^+^ populations are included as predictions in these files.
