# Supplementary material for: Detecting rare structural variation in evolving microbial populations from new sequence junctions using breseq
Source: Front Genet. 2015 Jan 21;5:468. doi: 10.3389/fgene.2014.00468 (PMC4301190; doi:10.3389/fgene.2014.00468)
Supplement: Data Sheet 1 — Genetic diversity predicted in the LTEE E. coli genomes. Full details of all polymorphisms predicted by breseq are provided in one merged HTML comparison table and in individual files for each population sample in Genome Diff format. In the comparison table, the delta symbol (Δ) indicates that the mutation in the specified row is fully contained within a region that is deleted within the specified sample. The machine-parsable Genome Diff file format is fully described in the breseq documentation. Note that the recD and araA mutations present in the ancestors of all Ara+ populations are included as predictions in these files. [file DataSheet1.ZIP › polymorphism_predictions/comparison_table.html]

Mutation Comparison


| Predicted mutations | | | | | | | | | | | | | | | | | | | | | | | | | | | | |
| --- | --- | --- | --- | --- | --- | --- | --- | --- | --- | --- | --- | --- | --- | --- | --- | --- | --- | --- | --- | --- | --- | --- | --- | --- | --- | --- | --- | --- |
| position | mutation | REL768 Ara+1 500 gen | REL958 Ara+1 1000 gen | REL769 Ara+2 500 gen | REL959 Ara+2 1000 gen | REL770 Ara+3 500 gen | REL960 Ara+3 1000 gen | REL771 Ara+4 500 gen | REL961 Ara+4 1000 gen | REL772 Ara+5 500 gen | REL962 Ara+5 1000 gen | REL773 Ara+6 500 gen | REL963 Ara+6 1000 gen | REL762 Ara−1 500 gen | REL964 Ara−1 1000 gen | REL763 Ara−2 500 gen | REL965 Ara−2 1000 gen | REL764 Ara−3 500 gen | REL966 Ara−3 1000 gen | REL765 Ara−4 500 gen | REL967 Ara−4 1000 gen | REL766 Ara−5 500 gen | REL968 Ara−5 1000 gen | REL767 Ara−6 500 gen | REL969 Ara−6 1000 gen | annotation | gene | description |
| 16,972 | IS*150* (+) |  |  |  |  |  |  |  |  |  |  |  |  |  |  |  | 6.2% |  |  |  |  |  |  |  |  | intergenic (‑14/‑517) | *mokC* ← / → *nhaA* | regulatory protein for HokC, overlaps CDS of hokC/pH‑dependent sodium/proton antiporter |
| 70,867 | T→C | 100% | 100% | 100% | 100% | 100% | 100% | 100% | 100% | 100% | 100% | 100% | 100% |  |  |  |  |  |  |  |  |  |  |  |  | D92G (GAC→GGC) | *araA* ← | L‑arabinose isomerase |
| 92,356 | C→T |  |  |  |  |  |  |  |  |  |  |  |  |  |  |  |  | 52.9% |  |  |  |  |  |  |  | intergenic (+520/‑82) | *fruR* → / → *yabB* | DNA‑binding transcriptional dual regulator/hypothetical protein |
| 92,385 | C→T |  |  |  |  |  |  |  |  |  |  |  |  |  |  |  |  |  |  |  |  |  |  | 24.5% |  | intergenic (+549/‑53) | *fruR* → / → *yabB* | DNA‑binding transcriptional dual regulator/hypothetical protein |
| 92,424 | IS*150* (+) +3 bp |  |  |  |  | 3.8% |  |  |  |  |  |  |  |  |  |  |  |  |  |  |  |  |  |  |  | intergenic (+588/‑12) | *fruR* → / → *yabB* | DNA‑binding transcriptional dual regulator/hypothetical protein |
| 92,424 | IS*150* (+) +4 bp |  |  |  |  |  |  |  |  | 3.7% | 8.1% |  |  |  |  | 24.0% |  | 6.2% |  |  |  |  |  |  |  | intergenic (+588/‑11) | *fruR* → / → *yabB* | DNA‑binding transcriptional dual regulator/hypothetical protein |
| 92,425 | IS*150* (+) +3 bp |  |  |  |  |  |  |  |  |  |  |  |  |  |  |  |  |  |  |  |  | 1.4% |  |  |  | intergenic (+589/‑11) | *fruR* → / → *yabB* | DNA‑binding transcriptional dual regulator/hypothetical protein |
| 92,427 | IS*150* (+) | 2.8% |  |  |  |  |  |  |  |  |  |  |  |  |  |  |  |  |  |  |  |  |  |  |  | intergenic (+591/‑12) | *fruR* → / → *yabB* | DNA‑binding transcriptional dual regulator/hypothetical protein |
| 92,876 | IS*150* (+) +3 bp |  |  |  |  |  |  |  |  |  |  |  |  |  |  |  |  | 5.3% | 100% |  |  |  |  |  |  | coding (439‑441/459 nt) | *yabB* → | hypothetical protein |
| 159,104 | Δ1 bp |  |  |  |  |  |  |  |  |  |  |  |  |  |  |  | 11.0% |  |  |  |  |  |  |  |  | intergenic (‑59/+46) | *ecpD* ← / ← *yadN* | predicted periplasmic pilin chaperone/predicted fimbrial‑like adhesin protein |
| position | mutation | REL768 Ara+1 500 gen | REL958 Ara+1 1000 gen | REL769 Ara+2 500 gen | REL959 Ara+2 1000 gen | REL770 Ara+3 500 gen | REL960 Ara+3 1000 gen | REL771 Ara+4 500 gen | REL961 Ara+4 1000 gen | REL772 Ara+5 500 gen | REL962 Ara+5 1000 gen | REL773 Ara+6 500 gen | REL963 Ara+6 1000 gen | REL762 Ara−1 500 gen | REL964 Ara−1 1000 gen | REL763 Ara−2 500 gen | REL965 Ara−2 1000 gen | REL764 Ara−3 500 gen | REL966 Ara−3 1000 gen | REL765 Ara−4 500 gen | REL967 Ara−4 1000 gen | REL766 Ara−5 500 gen | REL968 Ara−5 1000 gen | REL767 Ara−6 500 gen | REL969 Ara−6 1000 gen | annotation | gene | description |
| 162,788 | G→T |  |  |  |  |  |  |  |  |  |  |  |  |  |  |  |  |  |  |  |  |  |  |  | 5.2% | P59Q (CCG→CAG) | *yadB* ← | glutamyl‑Q tRNA(Asp) synthetase |
| 214,562 | C→A |  |  |  |  | 5.4% |  |  |  |  |  |  |  |  |  | 6.2% |  |  |  |  |  |  |  |  |  | S681Y (TCC→TAC) | *ldcC* → | lysine decarboxylase 2, constitutive |
| 215,053 | G→T |  | 5.3% |  | 5.8% | 6.5% |  | 5.7% |  |  |  |  | 5.9% | 6.7% |  | 8.2% |  |  |  |  |  | 5.2% |  |  |  | R112L (CGC→CTC) | *yaeR* → | predicted lyase |
| 250,487 | C→A |  |  |  |  |  |  |  |  | 5.2% |  |  |  |  |  |  |  |  |  |  |  |  |  |  |  | intergenic (‑111/+45) | *yafK* ← / ← *yafQ* | hypothetical protein/predicted toxin of the YafQ‑DinJ toxin‑antitoxin system |
| 319,840 | C→A |  |  |  |  |  |  |  |  |  |  |  |  |  | 6.4% |  |  |  |  |  |  |  |  |  |  | A418A (GCG→GCT) | *prpR* ← | DNA‑binding transcriptional activator |
| 360,117 | T→A |  |  |  |  |  |  |  |  |  |  |  |  |  |  |  |  |  |  |  |  | 5.1% | 16.3% |  |  | D320E (GAT→GAA) | *yaiT* → | conserved hypothetical protein |
| 362,354 | Δ1 bp |  |  |  |  |  |  |  |  |  |  |  | 11.5% |  |  |  |  |  |  |  |  |  |  |  |  | coding (248/669 nt) | *yaiV* → | predicted DNA‑binding transcriptional regulator |
| 427,741 | G→C |  |  |  |  |  | 6.3% |  |  |  |  |  |  |  |  |  |  |  |  |  |  |  |  |  |  | G120G (GGG→GGC) | *clpP* → | ATP‑dependent Clp protease proteolytic subunit |
| 474,213 | C→A |  |  |  |  |  |  |  |  |  |  |  |  |  |  |  |  |  |  |  |  | 5.9% |  |  |  | G365\* (GGA→TGA) | *ybaL* ← | predicted transporter with NAD(P)‑binding Rossmann‑fold domain |
| 474,383 | Δ9 bp |  |  |  |  | 26.6% | 94.6% |  |  |  |  |  |  |  |  |  |  |  |  |  |  |  |  |  |  | coding (915‑923/1677 nt) | *ybaL* ← | predicted transporter with NAD(P)‑binding Rossmann‑fold domain |
| position | mutation | REL768 Ara+1 500 gen | REL958 Ara+1 1000 gen | REL769 Ara+2 500 gen | REL959 Ara+2 1000 gen | REL770 Ara+3 500 gen | REL960 Ara+3 1000 gen | REL771 Ara+4 500 gen | REL961 Ara+4 1000 gen | REL772 Ara+5 500 gen | REL962 Ara+5 1000 gen | REL773 Ara+6 500 gen | REL963 Ara+6 1000 gen | REL762 Ara−1 500 gen | REL964 Ara−1 1000 gen | REL763 Ara−2 500 gen | REL965 Ara−2 1000 gen | REL764 Ara−3 500 gen | REL966 Ara−3 1000 gen | REL765 Ara−4 500 gen | REL967 Ara−4 1000 gen | REL766 Ara−5 500 gen | REL968 Ara−5 1000 gen | REL767 Ara−6 500 gen | REL969 Ara−6 1000 gen | annotation | gene | description |
| 475,185 | C→T |  |  |  |  |  |  |  |  |  |  |  |  |  |  |  |  |  |  |  |  |  | 9.5% |  |  | V41M (GTG→ATG) | *ybaL* ← | predicted transporter with NAD(P)‑binding Rossmann‑fold domain |
| 499,183 | IS*1* (–) +9 bp |  |  |  |  |  |  |  |  |  | 17.9% |  |  |  |  |  |  |  |  |  |  |  |  |  |  | coding (3756‑3764/4281 nt) | *rhsD* → | rhsD element protein |
| 561,126 | A→C |  | 14.7% |  |  |  |  |  |  |  |  |  |  |  |  |  |  |  |  |  |  |  |  |  |  | intergenic (‑142/+372) | *ompT* ← / ← *envY* | outer membrane protease VII (outer membrane protein 3b)/DNA‑binding transcriptional activator of porin biosynthesis |
| 583,589 | Δ1 bp |  |  |  |  |  |  |  |  |  |  |  |  |  |  |  |  |  |  |  | 5.8% |  |  |  |  | coding (728/1377 nt) | *pheP* → | phenylalanine transporter |
| 589,936 | IS*150* (–) |  |  |  |  |  |  |  |  |  |  |  |  |  |  |  |  |  |  |  |  |  |  | NA |  | coding (138‑139/504 nt) | *insB‑7* ← | IS1 protein insB |
| 648,665 | +AGA |  |  |  |  |  |  |  |  |  |  |  | 17.3% |  |  |  |  |  |  |  |  |  |  |  |  | coding (233/1113 nt) | *mrdB* ← | cell wall shape‑determining protein |
| 649,522 | C→T |  |  |  |  |  |  |  |  |  |  |  |  |  |  |  | 76.5% |  |  |  |  |  |  |  |  | G427D (GGC→GAC) | *mrdA* ← | transpeptidase involved in peptidoglycan synthesis (penicillin‑binding protein 2) |
| 650,281 | T→A |  |  |  |  |  |  |  |  |  |  |  | 5.6% |  |  |  |  |  |  |  |  |  |  |  |  | H174L (CAC→CTC) | *mrdA* ← | transpeptidase involved in peptidoglycan synthesis (penicillin‑binding protein 2) |
| 650,542 | T→G |  |  |  |  |  |  |  |  |  |  |  | 41.4% |  |  |  |  |  |  |  |  |  |  |  |  | Q87P (CAG→CCG) | *mrdA* ← | transpeptidase involved in peptidoglycan synthesis (penicillin‑binding protein 2) |
| 651,601 | T→A |  |  |  |  |  |  |  |  |  |  |  |  |  |  |  |  |  |  |  |  | 5.1% | 84.1% |  |  | Q7L (CAG→CTG) | *ybeB* ← | hypothetical protein |
| position | mutation | REL768 Ara+1 500 gen | REL958 Ara+1 1000 gen | REL769 Ara+2 500 gen | REL959 Ara+2 1000 gen | REL770 Ara+3 500 gen | REL960 Ara+3 1000 gen | REL771 Ara+4 500 gen | REL961 Ara+4 1000 gen | REL772 Ara+5 500 gen | REL962 Ara+5 1000 gen | REL773 Ara+6 500 gen | REL963 Ara+6 1000 gen | REL762 Ara−1 500 gen | REL964 Ara−1 1000 gen | REL763 Ara−2 500 gen | REL965 Ara−2 1000 gen | REL764 Ara−3 500 gen | REL966 Ara−3 1000 gen | REL765 Ara−4 500 gen | REL967 Ara−4 1000 gen | REL766 Ara−5 500 gen | REL968 Ara−5 1000 gen | REL767 Ara−6 500 gen | REL969 Ara−6 1000 gen | annotation | gene | description |
| 651,631 | T→A |  |  |  |  |  |  |  |  |  |  |  | 19.2% |  |  |  |  |  |  |  |  |  |  |  |  | intergenic (‑11/+249) | *ybeB* ← / ← *phpB* | hypothetical protein/predicted alpha‑ribazole‑5'‑P phosphatase |
| 659,015 | G→T |  |  |  |  |  |  |  |  |  |  |  | 6.4% |  |  |  |  |  |  |  |  |  |  |  |  | pseudogene (117/513 nt) | *ybeQ* ← | hypothetical protein; b0644\_2 |
| 824,453 | C→A |  |  |  |  |  |  |  |  |  |  |  |  | 6.2% |  |  |  |  |  |  |  |  |  |  |  | N319K (AAC→AAA) | *rhlE* → | RNA helicase |
| 920,514 | T→C |  |  |  |  |  |  |  |  |  |  |  |  |  |  |  |  |  | 100% |  |  |  |  |  |  | intergenic (‑73/+145) | *artP* ← / ← *ybjP* | arginine transporter subunit/predicted lipoprotein |
| 979,041 | Δ1 bp |  |  |  |  | 5.4% |  |  |  |  |  |  |  |  |  |  |  |  |  |  |  |  |  |  |  | intergenic (+65/‑46) | *cmk* → / → *rpsA* | cytidylate kinase/30S ribosomal protein S1 |
| 1,120,061 | IS*3* (+) |  |  |  |  |  |  |  |  |  |  |  |  |  |  |  |  |  |  |  | 11.6% |  |  |  |  | coding (78‑79/312 nt) | *ymdA* → | hypothetical protein |
| 1,196,977 | G→T |  |  |  |  |  |  |  |  |  |  |  |  |  |  |  |  |  | 5.2% |  |  |  |  |  |  | T40N (ACC→AAC) | *ymfA* ← | predicted inner membrane protein |
| 1,224,138 | C→A |  |  |  |  |  |  | 5.8% | 14.4% |  |  | 8.1% | 20.1% |  |  |  |  |  |  |  |  |  |  |  |  | intergenic (+70/+302) | *ycgI* → / ← *minE* | hypothetical protein/cell division topological specificity factor MinE |
| 1,230,390 | Δ1 bp |  |  |  |  |  |  |  |  |  |  |  |  |  |  |  |  |  |  |  |  |  |  | 5.3% |  | pseudogene (155/516 nt) | *hlyE* ← | frameshift; hemolysin E; b1182\_2 |
| 1,270,660 | IS*150* (–) |  |  |  |  |  |  |  |  |  |  |  |  |  |  |  |  | 3.1% |  |  |  |  |  |  |  | intergenic (‑167/+239) | *ldrC* ← / ← *chaA* | toxic polypeptide, small/calcium/sodium:proton antiporter |
| position | mutation | REL768 Ara+1 500 gen | REL958 Ara+1 1000 gen | REL769 Ara+2 500 gen | REL959 Ara+2 1000 gen | REL770 Ara+3 500 gen | REL960 Ara+3 1000 gen | REL771 Ara+4 500 gen | REL961 Ara+4 1000 gen | REL772 Ara+5 500 gen | REL962 Ara+5 1000 gen | REL773 Ara+6 500 gen | REL963 Ara+6 1000 gen | REL762 Ara−1 500 gen | REL964 Ara−1 1000 gen | REL763 Ara−2 500 gen | REL965 Ara−2 1000 gen | REL764 Ara−3 500 gen | REL966 Ara−3 1000 gen | REL765 Ara−4 500 gen | REL967 Ara−4 1000 gen | REL766 Ara−5 500 gen | REL968 Ara−5 1000 gen | REL767 Ara−6 500 gen | REL969 Ara−6 1000 gen | annotation | gene | description |
| 1,270,663 | IS*150* (–) |  |  |  |  |  |  |  |  |  |  |  |  | 2.8% |  |  |  |  |  |  |  |  |  |  |  | intergenic (‑170/+236) | *ldrC* ← / ← *chaA* | toxic polypeptide, small/calcium/sodium:proton antiporter |
| 1,270,663 | IS*150* (+) |  |  |  |  |  |  |  |  |  |  |  |  |  |  |  |  |  |  |  |  |  | 6.4% |  |  | intergenic (‑170/+236) | *ldrC* ← / ← *chaA* | toxic polypeptide, small/calcium/sodium:proton antiporter |
| 1,305,169 | C→A |  |  |  |  |  | 7.0% |  |  |  |  |  |  |  |  |  |  |  |  |  |  |  |  |  |  | A357A (GCG→GCT) | *cls* ← | cardiolipin synthetase |
| 1,329,435 | G→C | 63.4% | 90.7% |  |  |  |  |  |  |  |  |  |  |  |  |  |  |  |  |  |  |  |  |  |  | V6L (GTC→CTC) | *topA* → | DNA topoisomerase I |
| 1,329,516 | C→T |  |  |  |  |  |  |  |  |  |  |  |  | 53.9% | 40.0% |  |  |  |  |  |  |  |  |  |  | H33Y (CAC→TAC) | *topA* → | DNA topoisomerase I |
| 1,329,520 | T→G |  |  |  |  |  |  |  |  |  |  |  |  | 39.5% | 54.8% |  |  |  |  |  |  |  |  |  |  | I34S (ATC→AGC) | *topA* → | DNA topoisomerase I |
| 1,329,996 | G→A |  |  |  |  |  |  |  |  |  |  |  |  |  |  |  |  |  |  |  |  | 6.1% |  |  |  | A193T (GCC→ACC) | *topA* → | DNA topoisomerase I |
| 1,330,012 | C→T |  |  |  |  | 13.3% |  |  |  |  |  |  |  |  |  |  |  |  |  |  |  |  |  |  |  | S198L (TCG→TTG) | *topA* → | DNA topoisomerase I |
| 1,330,018 | C→A |  |  |  |  |  |  |  |  |  |  |  |  |  |  |  |  |  |  |  |  | 51.2% | 83.4% |  |  | A200E (GCG→GAG) | *topA* → | DNA topoisomerase I |
| 1,331,020 | T→A | 34.0% |  |  |  |  |  |  |  |  |  |  |  |  |  |  |  |  |  |  |  |  |  |  |  | F534Y (TTC→TAC) | *topA* → | DNA topoisomerase I |
| position | mutation | REL768 Ara+1 500 gen | REL958 Ara+1 1000 gen | REL769 Ara+2 500 gen | REL959 Ara+2 1000 gen | REL770 Ara+3 500 gen | REL960 Ara+3 1000 gen | REL771 Ara+4 500 gen | REL961 Ara+4 1000 gen | REL772 Ara+5 500 gen | REL962 Ara+5 1000 gen | REL773 Ara+6 500 gen | REL963 Ara+6 1000 gen | REL762 Ara−1 500 gen | REL964 Ara−1 1000 gen | REL763 Ara−2 500 gen | REL965 Ara−2 1000 gen | REL764 Ara−3 500 gen | REL966 Ara−3 1000 gen | REL765 Ara−4 500 gen | REL967 Ara−4 1000 gen | REL766 Ara−5 500 gen | REL968 Ara−5 1000 gen | REL767 Ara−6 500 gen | REL969 Ara−6 1000 gen | annotation | gene | description |
| 1,384,861 | C→T |  |  |  |  |  |  |  |  |  | 11.1% |  |  |  |  |  |  |  |  |  |  |  |  |  |  | R328C (CGC→TGC) | *ycjF* → | hypothetical protein |
| 1,462,251 | IS*150* (–) |  |  |  |  |  |  |  |  |  |  |  |  |  |  | 1.6% |  |  |  |  |  |  |  |  |  | intergenic (‑13/‑329) | *mokB* ← / → *trg* | regulatory peptide/methyl‑accepting chemotaxis protein III, ribose and galactose sensor receptor |
| 1,462,253 | IS*150* (–) |  |  |  |  |  |  |  |  |  |  |  |  | 1.5% |  |  |  | 2.7% |  |  |  |  |  |  |  | intergenic (‑15/‑327) | *mokB* ← / → *trg* | regulatory peptide/methyl‑accepting chemotaxis protein III, ribose and galactose sensor receptor |
| 1,462,254 | IS*150* (–) |  |  |  |  |  |  |  |  |  |  |  |  |  |  |  |  |  |  |  |  |  |  | 2.5% |  | intergenic (‑16/‑326) | *mokB* ← / → *trg* | regulatory peptide/methyl‑accepting chemotaxis protein III, ribose and galactose sensor receptor |
| 1,462,266 | IS*150* (+) +3 bp |  | 10.3% |  |  |  |  |  |  |  |  |  |  |  |  |  |  |  |  |  |  |  |  |  |  | intergenic (‑28/‑311) | *mokB* ← / → *trg* | regulatory peptide/methyl‑accepting chemotaxis protein III, ribose and galactose sensor receptor |
| 1,462,268 | IS*150* (+) | 1.5% |  |  |  |  |  |  |  |  |  |  |  |  |  |  |  |  |  |  |  |  |  |  |  | intergenic (‑30/‑312) | *mokB* ← / → *trg* | regulatory peptide/methyl‑accepting chemotaxis protein III, ribose and galactose sensor receptor |
| 1,496,693 | A→G | 16.3% |  |  |  |  |  |  |  |  |  |  |  |  |  |  |  |  |  |  |  |  |  |  |  | D112G (GAT→GGT) | *yncG* → | predicted enzyme |
| 1,541,463 | C→A |  | 11.4% |  |  |  |  |  |  |  |  |  |  |  |  |  |  |  |  |  |  |  |  |  |  | G438C (GGC→TGC) | *yddW* ← | predicted liprotein |
| 1,567,432 | Δ1 bp |  |  |  |  |  |  |  |  |  |  |  |  |  |  |  |  |  | 8.7% |  |  |  |  |  |  | coding (1312/1323 nt) | *hipA* ← | regulator with hipB |
| 1,610,312 | IS*3* (–) +3 bp |  |  |  |  |  |  |  |  |  |  |  |  |  |  |  |  | 2.4% |  |  |  |  |  |  |  | intergenic (‑11/+135) | *ynfN* ← / ← *ECB\_01516* | predicted protein/putative cold‑shock protein |
| position | mutation | REL768 Ara+1 500 gen | REL958 Ara+1 1000 gen | REL769 Ara+2 500 gen | REL959 Ara+2 1000 gen | REL770 Ara+3 500 gen | REL960 Ara+3 1000 gen | REL771 Ara+4 500 gen | REL961 Ara+4 1000 gen | REL772 Ara+5 500 gen | REL962 Ara+5 1000 gen | REL773 Ara+6 500 gen | REL963 Ara+6 1000 gen | REL762 Ara−1 500 gen | REL964 Ara−1 1000 gen | REL763 Ara−2 500 gen | REL965 Ara−2 1000 gen | REL764 Ara−3 500 gen | REL966 Ara−3 1000 gen | REL765 Ara−4 500 gen | REL967 Ara−4 1000 gen | REL766 Ara−5 500 gen | REL968 Ara−5 1000 gen | REL767 Ara−6 500 gen | REL969 Ara−6 1000 gen | annotation | gene | description |
| 1,699,818 | Δ3 bp |  |  |  |  |  |  |  |  |  |  |  |  |  |  |  |  |  |  | 14.7% |  |  |  |  |  | coding (504‑506/2013 nt) | *ydhK* → | conserved inner membrane protein |
| 1,732,806 | G→A |  |  |  |  |  |  |  |  |  | 6.4% |  |  |  |  |  |  |  |  |  |  |  |  |  |  | intergenic (‑398/‑159) | *ydhZ* ← / → *pykF* | hypothetical protein/pyruvate kinase |
| 1,733,066 | C→G |  |  |  |  |  |  |  |  |  | 8.5% |  |  |  |  |  |  |  |  |  |  |  |  |  |  | N34K (AAC→AAG) | *pykF* → | pyruvate kinase |
| 1,733,141 | IS*150* (+) +3 bp |  |  |  |  |  |  |  |  |  |  |  |  |  |  |  |  |  | 100% |  |  |  |  |  |  | coding (177‑179/1413 nt) | *pykF* → | pyruvate kinase |
| 1,733,172 | C→A |  |  |  |  |  |  |  |  |  |  | 100% | 100% |  |  |  |  |  |  |  |  |  |  |  |  | P70T (CCG→ACG) | *pykF* → | pyruvate kinase |
| 1,733,173 | C→A |  |  |  |  |  | 81.4% |  |  |  |  |  |  |  |  |  |  |  |  |  |  |  |  |  |  | P70Q (CCG→CAG) | *pykF* → | pyruvate kinase |
| 1,733,343 | G→A |  |  |  |  |  |  |  |  |  |  |  |  |  |  |  |  | 9.9% |  |  |  |  |  |  |  | D127N (GAT→AAT) | *pykF* → | pyruvate kinase |
| 1,733,447 | Δ1 bp |  |  |  |  |  |  | 60.3% | 100% |  |  |  |  |  |  |  |  |  |  |  |  |  |  |  |  | coding (483/1413 nt) | *pykF* → | pyruvate kinase |
| 1,733,497 | T→G |  |  |  |  | 54.2% | 11.4% |  |  |  |  |  |  |  |  |  |  |  |  |  |  |  |  |  |  | L178R (CTG→CGG) | *pykF* → | pyruvate kinase |
| 1,733,511 | Δ183 bp |  |  |  |  |  |  |  |  |  |  | 2.2% |  |  |  |  |  |  |  |  |  |  |  |  |  | coding (547‑729/1413 nt) | *pykF* → | pyruvate kinase |
| position | mutation | REL768 Ara+1 500 gen | REL958 Ara+1 1000 gen | REL769 Ara+2 500 gen | REL959 Ara+2 1000 gen | REL770 Ara+3 500 gen | REL960 Ara+3 1000 gen | REL771 Ara+4 500 gen | REL961 Ara+4 1000 gen | REL772 Ara+5 500 gen | REL962 Ara+5 1000 gen | REL773 Ara+6 500 gen | REL963 Ara+6 1000 gen | REL762 Ara−1 500 gen | REL964 Ara−1 1000 gen | REL763 Ara−2 500 gen | REL965 Ara−2 1000 gen | REL764 Ara−3 500 gen | REL966 Ara−3 1000 gen | REL765 Ara−4 500 gen | REL967 Ara−4 1000 gen | REL766 Ara−5 500 gen | REL968 Ara−5 1000 gen | REL767 Ara−6 500 gen | REL969 Ara−6 1000 gen | annotation | gene | description |
| 1,733,559 | (TAAGCGTTCTG)1→2 |  |  |  |  |  |  |  |  |  |  | Δ |  |  |  | 3.5% |  |  |  |  |  |  |  |  |  | coding (595/1413 nt) | *pykF* → | pyruvate kinase |
| 1,733,640 | G→T |  |  |  |  |  |  |  |  |  |  | Δ |  |  |  |  |  |  |  |  |  |  | 5.2% |  |  | G226C (GGC→TGC) | *pykF* → | pyruvate kinase |
| 1,733,646 | +GTC :: IS*150* (–) |  |  |  |  |  |  |  |  |  |  | Δ |  |  |  |  |  |  |  |  |  |  | 12.3% |  |  | coding (681‑682/1413 nt) | *pykF* → | pyruvate kinase |
| 1,733,646 | IS*150* (–) +2 bp |  |  |  |  |  |  |  |  |  |  | Δ |  |  |  |  |  |  |  |  |  | 4.7% |  |  |  | coding (682‑683/1413 nt) | *pykF* → | pyruvate kinase |
| 1,733,647 | IS*RS011* (–) |  |  |  |  |  |  |  |  |  |  | Δ |  |  |  |  |  |  |  | 1.9% |  |  |  |  |  | coding (682‑683/1413 nt) | *pykF* → | pyruvate kinase |
| 1,733,685 | A→G |  |  |  |  |  |  |  |  |  |  | Δ |  |  | 14.0% |  |  |  |  |  |  |  |  |  |  | M241V (ATG→GTG) | *pykF* → | pyruvate kinase |
| 1,733,687 | G→A |  |  |  |  |  |  |  |  |  |  | Δ |  |  |  |  |  |  |  |  |  | 7.5% |  |  |  | M241I (ATG→ATA) | *pykF* → | pyruvate kinase |
| 1,733,752 | T→C |  |  |  |  |  |  |  |  |  |  |  |  |  |  |  |  |  |  |  |  |  | 6.4% |  |  | M263T (ATG→ACG) | *pykF* → | pyruvate kinase |
| 1,733,754 | A→T |  |  |  |  |  |  |  |  |  |  |  |  |  |  |  | 100% |  |  |  |  |  |  |  |  | I264F (ATC→TTC) | *pykF* → | pyruvate kinase |
| 1,733,865 | G→A |  |  |  |  |  |  |  |  |  |  |  |  |  |  |  |  |  |  |  |  |  |  | 64.2% | 100% | A301T (GCC→ACC) | *pykF* → | pyruvate kinase |
| position | mutation | REL768 Ara+1 500 gen | REL958 Ara+1 1000 gen | REL769 Ara+2 500 gen | REL959 Ara+2 1000 gen | REL770 Ara+3 500 gen | REL960 Ara+3 1000 gen | REL771 Ara+4 500 gen | REL961 Ara+4 1000 gen | REL772 Ara+5 500 gen | REL962 Ara+5 1000 gen | REL773 Ara+6 500 gen | REL963 Ara+6 1000 gen | REL762 Ara−1 500 gen | REL964 Ara−1 1000 gen | REL763 Ara−2 500 gen | REL965 Ara−2 1000 gen | REL764 Ara−3 500 gen | REL966 Ara−3 1000 gen | REL765 Ara−4 500 gen | REL967 Ara−4 1000 gen | REL766 Ara−5 500 gen | REL968 Ara−5 1000 gen | REL767 Ara−6 500 gen | REL969 Ara−6 1000 gen | annotation | gene | description |
| 1,733,865 | G→T |  | 5.5% |  |  |  |  |  |  |  | 5.9% |  |  |  |  |  |  |  |  |  |  |  |  |  |  | A301S (GCC→TCC) | *pykF* → | pyruvate kinase |
| 1,734,102 | G→A |  |  |  |  |  |  |  |  |  | 7.8% |  |  |  |  |  |  |  |  |  |  |  |  |  |  | G380S (GGC→AGC) | *pykF* → | pyruvate kinase |
| 1,734,106 | G→C |  |  | 100% | 100% |  |  |  |  |  |  |  |  |  |  |  |  |  |  |  |  |  |  |  |  | G381A (GGT→GCT) | *pykF* → | pyruvate kinase |
| 1,734,349 | C→T |  |  |  |  |  |  |  |  |  |  |  |  |  |  |  |  |  |  | 100% | 100% |  |  |  |  | T462I (ACT→ATT) | *pykF* → | pyruvate kinase |
| 1,776,434 | IS*150* (–) +4 bp |  |  |  |  |  | 18.6% |  |  |  |  |  |  |  |  |  |  |  |  |  |  |  |  |  |  | intergenic (‑225/+56) | *pheS* ← / ← *pheM* | phenylalanyl‑tRNA synthetase alpha subunit/phenylalanyl‑tRNA synthetase operon leader peptide |
| 2,020,131 | IS*150* (+) +3 bp |  |  |  |  |  |  |  |  |  |  |  |  |  |  |  |  | 7.4% |  |  |  |  |  |  |  | intergenic (+83/‑61) | *hisL* → / → *hisG* | his operon leader peptide/ATP phosphoribosyltransferase |
| 2,020,133 | IS*150* (–) |  |  |  |  |  | 5.7% |  |  |  |  |  |  |  |  |  |  |  |  |  |  |  |  |  |  | intergenic (+85/‑62) | *hisL* → / → *hisG* | his operon leader peptide/ATP phosphoribosyltransferase |
| 2,028,516 | C→A |  |  |  |  | 6.8% |  |  |  | 8.2% |  |  |  |  |  | 5.7% |  |  | 5.7% |  |  |  |  |  |  | R367L (CGT→CTT) | *ugd* ← | UDP‑glucose 6‑dehydrogenase |
| 2,035,102 | C→A |  |  |  |  |  |  |  |  |  |  |  |  |  |  |  |  |  |  |  |  |  | 5.8% |  |  | R252M (AGG→ATG) | *wbbD* ← | putative glycosyltransferase WbbD |
| 2,035,870 | Δ1 bp |  |  |  |  | 6.2% |  |  |  |  |  |  |  |  |  |  |  |  |  |  |  |  |  |  |  | coding (1089/1113 nt) | *wbbC* ← | putative glycosyltransferase WbbC |
| position | mutation | REL768 Ara+1 500 gen | REL958 Ara+1 1000 gen | REL769 Ara+2 500 gen | REL959 Ara+2 1000 gen | REL770 Ara+3 500 gen | REL960 Ara+3 1000 gen | REL771 Ara+4 500 gen | REL961 Ara+4 1000 gen | REL772 Ara+5 500 gen | REL962 Ara+5 1000 gen | REL773 Ara+6 500 gen | REL963 Ara+6 1000 gen | REL762 Ara−1 500 gen | REL964 Ara−1 1000 gen | REL763 Ara−2 500 gen | REL965 Ara−2 1000 gen | REL764 Ara−3 500 gen | REL966 Ara−3 1000 gen | REL765 Ara−4 500 gen | REL967 Ara−4 1000 gen | REL766 Ara−5 500 gen | REL968 Ara−5 1000 gen | REL767 Ara−6 500 gen | REL969 Ara−6 1000 gen | annotation | gene | description |
| 2,048,103 | IS*1* (+) +9 bp |  |  | 20.2% | 80.4% |  |  |  |  |  |  |  |  |  |  |  |  |  |  |  |  |  |  |  |  | coding (1017‑1025/1395 nt) | *wcaM* ← | predicted colanic acid biosynthesis protein |
| 2,083,003 | C→T |  |  |  |  |  | 5.4% |  |  |  |  |  |  |  |  |  |  |  |  |  |  |  |  |  |  | R388Q (CGG→CAG) | *yegI* ← | hypothetical protein |
| 2,086,209 | IS*150* (+) +3 bp |  |  |  |  |  |  |  | 21.7% |  |  |  |  |  |  |  |  |  |  |  |  |  |  |  |  | intergenic (‑630/‑913) | *yegL* ← / → *yegM* | hypothetical protein/multidrug efflux system, subunit A |
| 2,093,027 | IS*150* (–) |  | 2.1% |  |  |  |  |  |  |  |  |  |  |  |  |  |  |  |  |  |  |  |  |  |  | coding (1533‑1534/3078 nt) | *yegO* → | multidrug efflux system, subunit C |
| 2,103,918 | (CCAG)7→8 |  |  |  |  |  |  |  |  |  |  |  |  |  |  | 97.3% | 92.9% | 56.6% |  |  |  |  |  |  |  | coding (185/216 nt) | *ECB\_01992* → | hypothetical protein |
| 2,205,480 | IS*150* (–) +4 bp |  |  |  |  |  |  |  | 9.4% |  |  |  |  |  |  |  |  |  |  |  |  |  |  |  |  | coding (891‑894/942 nt) | *rihB* ← | ribonucleoside hydrolase 2 |
| 2,221,442 | C→A |  |  |  |  |  |  |  |  |  |  |  |  |  |  |  |  |  | 6.7% |  |  |  |  |  |  | I92I (ATC→ATA) | *spr* → | predicted peptidase, outer membrane lipoprotein |
| 2,267,890 | C→T |  |  |  |  |  |  |  |  |  |  |  |  |  | 9.8% |  |  |  |  |  |  |  |  |  |  | A383V (GCA→GTA) | *atoS* → | sensory histidine kinase in two‑component regulatory system with AtoC |
| 2,275,076 | T→G |  |  |  |  |  |  |  |  |  |  |  |  |  |  |  |  |  |  |  |  | 100% | 100% |  |  | Q474H (CAA→CAC) | *yfaQ* ← | hypothetical protein |
| 2,448,493 | IS*186* (+) +6 bp |  |  |  |  |  |  |  |  |  |  |  |  |  |  |  |  |  |  |  |  |  | 12.0% |  |  | intergenic (+24/+21) | *nupC* → / ← *yfeA* | nucleoside (except guanosine) transporter/predicted diguanylate cyclase |
| position | mutation | REL768 Ara+1 500 gen | REL958 Ara+1 1000 gen | REL769 Ara+2 500 gen | REL959 Ara+2 1000 gen | REL770 Ara+3 500 gen | REL960 Ara+3 1000 gen | REL771 Ara+4 500 gen | REL961 Ara+4 1000 gen | REL772 Ara+5 500 gen | REL962 Ara+5 1000 gen | REL773 Ara+6 500 gen | REL963 Ara+6 1000 gen | REL762 Ara−1 500 gen | REL964 Ara−1 1000 gen | REL763 Ara−2 500 gen | REL965 Ara−2 1000 gen | REL764 Ara−3 500 gen | REL966 Ara−3 1000 gen | REL765 Ara−4 500 gen | REL967 Ara−4 1000 gen | REL766 Ara−5 500 gen | REL968 Ara−5 1000 gen | REL767 Ara−6 500 gen | REL969 Ara−6 1000 gen | annotation | gene | description |
| 2,456,191 | C→A |  | 7.1% |  |  |  |  |  |  |  |  |  |  |  |  | 6.9% |  |  |  |  |  |  |  |  |  | P174T (CCG→ACG) | *yfeH* → | predicted inner membrane protein |
| 2,622,101 | Δ1 bp |  |  |  |  |  |  | 5.3% |  |  |  |  |  |  |  |  |  |  |  |  |  |  |  |  |  | intergenic (+686/+9) | *yfhL* → / ← *acpS* | predicted 4Fe‑4S cluster‑containing protein/4'‑phosphopantetheinyl transferase |
| 2,713,428 | IS*186* (+) |  |  |  |  |  |  |  |  | 3.8% |  |  |  |  |  |  |  |  |  |  |  |  |  |  |  | noncoding (77‑78/93 nt) | *serV* ← | tRNA‑Ser |
| 2,720,615 | C→A |  |  |  |  |  |  |  |  |  |  |  |  |  |  |  |  |  |  |  | 8.6% |  |  |  |  | intergenic (‑180/‑76) | *mltB* ← / → *srlA* | membrane‑bound lytic murein transglycosylase B/glucitol/sorbitol‑specific enzyme IIC component of PTS |
| 2,802,598 | C→T |  |  | 5.0% |  |  |  |  |  |  |  |  |  |  |  |  |  |  |  |  |  |  |  |  |  | intergenic (‑19/+29) | *relA* ← / ← *rumA* | (p)ppGpp synthetase I/GTP pyrophosphokinase/23S rRNA (uracil‑5‑)‑methyltransferase |
| 2,802,769 | A→C |  |  |  |  |  |  |  |  |  |  |  |  |  |  |  |  |  |  |  |  |  |  | 18.2% | 8.0% | V387G (GTA→GGA) | *rumA* ← | 23S rRNA (uracil‑5‑)‑methyltransferase |
| 2,809,379 | G→T |  | 5.7% |  |  | 5.2% |  |  |  |  |  |  |  |  |  | 10.0% |  |  |  |  |  | 5.1% |  |  |  | G99G (GGC→GGA) | *gudX* ← | predicted glucarate dehydratase |
| 2,824,389 | IS*1* (–) |  |  |  |  |  |  |  |  |  |  |  |  |  |  |  |  |  |  |  |  | 1.8% |  |  |  | coding (83‑84/945 nt) | *ECB\_02648* ← | L‑fuculose phosphate aldolase |
| 2,826,327 | G→T |  |  |  |  |  |  |  |  |  |  |  |  |  |  |  |  |  |  | 7.0% |  |  |  |  |  | Q324K (CAA→AAA) | *ECB\_02650* ← | D‑ribose‑binding periplasmic protein |
| 2,847,052 | A→G | 100% | 100% | 100% | 100% | 100% | 100% | 100% | 100% | 100% | 100% | 100% | 100% |  |  |  |  |  |  |  |  |  |  |  |  | V10A (GTT→GCT) | *recD* ← | exonuclease V (RecBCD complex), alpha chain |
| position | mutation | REL768 Ara+1 500 gen | REL958 Ara+1 1000 gen | REL769 Ara+2 500 gen | REL959 Ara+2 1000 gen | REL770 Ara+3 500 gen | REL960 Ara+3 1000 gen | REL771 Ara+4 500 gen | REL961 Ara+4 1000 gen | REL772 Ara+5 500 gen | REL962 Ara+5 1000 gen | REL773 Ara+6 500 gen | REL963 Ara+6 1000 gen | REL762 Ara−1 500 gen | REL964 Ara−1 1000 gen | REL763 Ara−2 500 gen | REL965 Ara−2 1000 gen | REL764 Ara−3 500 gen | REL966 Ara−3 1000 gen | REL765 Ara−4 500 gen | REL967 Ara−4 1000 gen | REL766 Ara−5 500 gen | REL968 Ara−5 1000 gen | REL767 Ara−6 500 gen | REL969 Ara−6 1000 gen | annotation | gene | description |
| 2,871,889 | G→A |  |  |  |  |  |  |  |  |  |  |  |  |  |  |  |  |  |  |  |  |  | 8.4% |  |  | G224S (GGC→AGC) | *galR* → | DNA‑binding transcriptional repressor |
| 3,080,114 | IS*150* (–) +3 bp |  | 12.0% |  |  |  |  |  |  |  |  |  |  |  |  |  |  |  |  |  |  |  |  |  |  | pseudogene (119‑121/1005 nt) | *hybO* ← | hydrogenase‑2, small chain; b2997\_1 |
| 3,162,266 | C→A |  |  |  |  |  |  |  |  |  |  |  |  |  | 6.6% |  |  |  |  |  |  |  |  |  |  | R188S (CGC→AGC) | *ygjK* → | predicted glycosyl hydrolase |
| 3,250,883 | IS*150* (–) +3 bp |  |  |  |  |  |  |  |  |  |  |  |  |  |  |  |  |  |  |  |  |  |  | 2.2% |  | coding (364‑366/2673 nt) | *infB* ← | translation initiation factor IF‑2 |
| 3,250,993 | G→A |  |  |  | 20.7% |  |  |  |  |  |  |  |  |  |  |  |  |  |  |  |  |  |  |  |  | R86C (CGC→TGC) | *infB* ← | translation initiation factor IF‑2 |
| 3,327,004 | G→T |  |  |  |  |  |  |  |  |  |  |  |  |  |  |  |  |  |  |  |  |  |  |  | 86.3% | A289D (GCT→GAT) | *mreC* ← | cell wall structural complex MreBCD transmembrane component MreC |
| 3,327,538 | G→A |  |  |  |  |  |  |  |  |  |  |  |  |  |  |  |  |  |  |  | 32.1% |  |  |  |  | P111L (CCG→CTG) | *mreC* ← | cell wall structural complex MreBCD transmembrane component MreC |
| 3,327,572 | C→T |  |  |  |  | 10.5% | 84.4% |  |  |  |  |  |  |  |  |  |  |  |  |  |  |  |  |  |  | E100K (GAG→AAG) | *mreC* ← | cell wall structural complex MreBCD transmembrane component MreC |
| 3,328,012 | T→C |  |  |  |  |  |  |  |  |  |  |  |  |  |  |  |  |  |  |  | 6.4% |  |  |  |  | T323A (ACC→GCC) | *mreB* ← | cell wall structural complex MreBCD, actin‑like component MreB |
| 3,328,402 | G→A |  |  |  |  |  |  |  |  | 85.1% | 53.2% |  |  |  |  |  |  |  |  |  |  |  |  |  |  | R193C (CGT→TGT) | *mreB* ← | cell wall structural complex MreBCD, actin‑like component MreB |
| position | mutation | REL768 Ara+1 500 gen | REL958 Ara+1 1000 gen | REL769 Ara+2 500 gen | REL959 Ara+2 1000 gen | REL770 Ara+3 500 gen | REL960 Ara+3 1000 gen | REL771 Ara+4 500 gen | REL961 Ara+4 1000 gen | REL772 Ara+5 500 gen | REL962 Ara+5 1000 gen | REL773 Ara+6 500 gen | REL963 Ara+6 1000 gen | REL762 Ara−1 500 gen | REL964 Ara−1 1000 gen | REL763 Ara−2 500 gen | REL965 Ara−2 1000 gen | REL764 Ara−3 500 gen | REL966 Ara−3 1000 gen | REL765 Ara−4 500 gen | REL967 Ara−4 1000 gen | REL766 Ara−5 500 gen | REL968 Ara−5 1000 gen | REL767 Ara−6 500 gen | REL969 Ara−6 1000 gen | annotation | gene | description |
| 3,328,416 | C→A |  |  |  |  |  |  |  |  |  |  |  |  |  |  |  |  |  |  |  |  | 8.9% |  |  |  | R188L (CGC→CTC) | *mreB* ← | cell wall structural complex MreBCD, actin‑like component MreB |
| 3,328,619 | C→A |  |  |  | 57.3% |  |  |  |  |  |  |  |  |  |  |  |  |  |  |  |  |  |  |  |  | Q120H (CAG→CAT) | *mreB* ← | cell wall structural complex MreBCD, actin‑like component MreB |
| 3,328,903 | C→T |  |  |  |  |  |  |  |  |  |  |  |  |  |  |  |  |  |  |  | 11.9% |  |  |  |  | V26I (GTA→ATA) | *mreB* ← | cell wall structural complex MreBCD, actin‑like component MreB |
| 3,371,734 | A→C |  |  |  |  |  |  |  |  |  |  |  |  |  |  |  |  |  |  |  |  |  |  |  | 18.4% | I90S (ATC→AGC) | *prlA* ← | protein translocase subunit SecY |
| 3,427,811 | C→A |  |  |  |  |  |  |  |  |  |  |  |  |  |  |  |  |  |  |  | 5.0% |  |  |  |  | intergenic (+291/‑4) | *yhfL* → / → *yhfM* | conserved secreted peptide/predicted fructoselysine transporter |
| 3,475,057 | C→T |  |  |  |  |  |  |  |  |  |  |  |  |  |  |  |  |  |  |  | 8.5% |  |  |  |  | intergenic (+193/‑168) | *yhgI* → / → *gntT* | predicted gluconate transport associated protein/gluconate transporter, high‑affinity GNT I system |
| 3,581,976 | A→G |  |  |  |  |  |  |  |  |  |  |  |  |  |  |  |  |  |  |  |  | 5.6% |  |  |  | intergenic (+624/‑5) | *arsC* → / → *yhiS* | arsenate reductase/hypothetical protein |
| 3,600,272 | G→T |  | 6.6% |  |  |  |  |  |  |  |  |  |  |  |  | 9.3% |  |  |  |  |  |  |  |  |  | E397\* (GAA→TAA) | *treF* → | cytoplasmic trehalase |
| 3,667,119 | Δ3 bp |  |  |  |  |  |  |  |  |  |  | 11.2% |  |  |  |  |  |  |  |  |  |  |  |  |  | coding (513‑515/825 nt) | *bax* ← | hypothetical protein |
| 3,712,866 | G→T |  |  |  |  |  |  |  |  |  |  |  | 6.8% |  |  |  |  |  |  |  |  |  |  |  |  | L201L (CTG→CTT) | *ECB\_03459* → | conserved hypothetical protein |
| position | mutation | REL768 Ara+1 500 gen | REL958 Ara+1 1000 gen | REL769 Ara+2 500 gen | REL959 Ara+2 1000 gen | REL770 Ara+3 500 gen | REL960 Ara+3 1000 gen | REL771 Ara+4 500 gen | REL961 Ara+4 1000 gen | REL772 Ara+5 500 gen | REL962 Ara+5 1000 gen | REL773 Ara+6 500 gen | REL963 Ara+6 1000 gen | REL762 Ara−1 500 gen | REL964 Ara−1 1000 gen | REL763 Ara−2 500 gen | REL965 Ara−2 1000 gen | REL764 Ara−3 500 gen | REL966 Ara−3 1000 gen | REL765 Ara−4 500 gen | REL967 Ara−4 1000 gen | REL766 Ara−5 500 gen | REL968 Ara−5 1000 gen | REL767 Ara−6 500 gen | REL969 Ara−6 1000 gen | annotation | gene | description |
| 3,761,376 | G→A |  |  |  |  |  |  |  |  |  |  |  |  |  |  |  |  |  |  |  |  | 5.9% |  |  |  | G207D (GGC→GAC) | *spoT* → | bifunctional (p)ppGpp synthetase II/ guanosine‑3',5'‑bis pyrophosphate 3'‑pyrophosphohydrolase |
| 3,761,376 | G→T |  |  |  |  | 58.2% |  |  |  |  |  |  |  |  |  |  |  |  |  |  |  |  |  |  |  | G207V (GGC→GTC) | *spoT* → | bifunctional (p)ppGpp synthetase II/ guanosine‑3',5'‑bis pyrophosphate 3'‑pyrophosphohydrolase |
| 3,761,382 | G→A |  |  |  |  |  |  | 90.7% | 100% |  |  |  |  |  |  |  |  |  |  |  |  |  |  |  |  | R209H (CGT→CAT) | *spoT* → | bifunctional (p)ppGpp synthetase II/ guanosine‑3',5'‑bis pyrophosphate 3'‑pyrophosphohydrolase |
| 3,761,922 | A→G |  |  |  |  |  |  |  |  |  |  |  |  |  |  |  |  |  |  | 100% | 100% |  |  |  |  | Y389C (TAC→TGC) | *spoT* → | bifunctional (p)ppGpp synthetase II/ guanosine‑3',5'‑bis pyrophosphate 3'‑pyrophosphohydrolase |
| 3,762,117 | A→T |  |  |  |  |  |  |  |  |  |  | 100% | 100% |  |  |  |  |  |  |  |  |  |  |  |  | N454I (AAT→ATT) | *spoT* → | bifunctional (p)ppGpp synthetase II/ guanosine‑3',5'‑bis pyrophosphate 3'‑pyrophosphohydrolase |
| 3,762,120 | C→A |  |  |  |  |  |  |  |  |  |  |  |  |  |  | 66.6% | 100% |  |  |  |  |  |  |  |  | A455D (GCC→GAC) | *spoT* → | bifunctional (p)ppGpp synthetase II/ guanosine‑3',5'‑bis pyrophosphate 3'‑pyrophosphohydrolase |
| 3,762,480 | G→T |  |  | 100% | 100% |  |  |  |  |  |  |  |  |  |  |  |  |  |  |  |  |  |  |  |  | R575L (CGC→CTC) | *spoT* → | bifunctional (p)ppGpp synthetase II/ guanosine‑3',5'‑bis pyrophosphate 3'‑pyrophosphohydrolase |
| 3,762,713 | A→C |  |  |  |  |  |  |  |  |  |  |  |  |  |  |  |  |  |  |  |  | 7.2% | 21.4% |  |  | N653H (AAT→CAT) | *spoT* → | bifunctional (p)ppGpp synthetase II/ guanosine‑3',5'‑bis pyrophosphate 3'‑pyrophosphohydrolase |
| 3,762,741 | A→T |  |  |  |  |  |  |  |  |  |  |  |  |  | 38.2% |  |  |  |  |  |  |  |  |  |  | K662I (AAA→ATA) | *spoT* → | bifunctional (p)ppGpp synthetase II/ guanosine‑3',5'‑bis pyrophosphate 3'‑pyrophosphohydrolase |
| 3,816,712 | Δ1 bp |  |  |  |  |  |  |  |  |  |  |  |  |  |  |  | 6.5% |  |  |  |  |  |  |  |  | coding (366/450 nt) | *yidI* → | predicted inner membrane protein |
| position | mutation | REL768 Ara+1 500 gen | REL958 Ara+1 1000 gen | REL769 Ara+2 500 gen | REL959 Ara+2 1000 gen | REL770 Ara+3 500 gen | REL960 Ara+3 1000 gen | REL771 Ara+4 500 gen | REL961 Ara+4 1000 gen | REL772 Ara+5 500 gen | REL962 Ara+5 1000 gen | REL773 Ara+6 500 gen | REL963 Ara+6 1000 gen | REL762 Ara−1 500 gen | REL964 Ara−1 1000 gen | REL763 Ara−2 500 gen | REL965 Ara−2 1000 gen | REL764 Ara−3 500 gen | REL966 Ara−3 1000 gen | REL765 Ara−4 500 gen | REL967 Ara−4 1000 gen | REL766 Ara−5 500 gen | REL968 Ara−5 1000 gen | REL767 Ara−6 500 gen | REL969 Ara−6 1000 gen | annotation | gene | description |
| 3,869,962 | Δ11 bp |  |  |  |  |  |  |  |  |  |  |  |  |  |  |  |  |  |  |  |  | 2.1% |  |  |  | coding (794‑804/960 nt) | *pstC* ← | phosphate transporter subunit |
| 3,887,214 | T→C |  |  |  |  |  |  |  |  |  |  |  | 10.6% |  |  |  |  |  |  |  |  |  |  |  |  | I51V (ATC→GTC) | *asnC* ← | DNA‑binding transcriptional dual regulator |
| 3,893,549 | IS*150* (–) |  |  | NA |  |  |  |  |  |  |  |  |  |  |  |  |  |  |  |  |  |  |  |  |  | intergenic (+4/‑53) | *kup* → / → *insJ‑5* | potassium transporter/IS150 hypothetical protein |
| 3,893,550 | +G |  |  |  |  |  |  |  |  |  |  |  |  |  |  |  |  |  |  |  |  |  |  |  | 18.8% | intergenic (+5/‑51) | *kup* → / → *insJ‑5* | potassium transporter/IS150 hypothetical protein |
| 3,894,997 | Δ7,813 bp |  |  |  |  |  |  |  |  |  |  |  |  |  |  |  |  |  |  |  |  |  |  | 1.7% |  |  | *rbsD*–*[yieP]* | *rbsD, rbsA, rbsC, rbsB, rbsK, rbsR, yieO, [yieP]* |
| 3,894,997 | Δ7,552 bp |  |  |  |  |  |  |  |  |  |  |  |  |  |  |  |  | 2.4% |  |  |  |  |  | Δ |  |  | *rbsD*–*[yieP]* | *rbsD, rbsA, rbsC, rbsB, rbsK, rbsR, yieO, [yieP]* |
| 3,894,997 | Δ7,131 bp |  |  |  |  |  |  |  |  |  |  |  |  |  |  |  |  | Δ |  |  |  | 0.94% |  | Δ |  |  | *rbsD*–*[yieO]* | *rbsD, rbsA, rbsC, rbsB, rbsK, rbsR, [yieO]* |
| 3,894,997 | Δ6,934 bp |  |  |  |  |  |  |  |  |  |  |  |  | 61.1% | 41.5% |  |  | Δ |  |  |  | Δ |  | Δ |  |  | *rbsD*–*[yieO]* | *rbsD, rbsA, rbsC, rbsB, rbsK, rbsR, [yieO]* |
| 3,894,997 | Δ6,712 bp |  |  |  |  |  |  |  |  |  |  |  |  | Δ | Δ |  |  | Δ |  |  |  | Δ |  | 3.8% | 91.8% |  | *rbsD*–*[yieO]* | *rbsD, rbsA, rbsC, rbsB, rbsK, rbsR, [yieO]* |
| 3,894,997 | Δ6,626 bp |  |  |  |  |  |  |  |  |  |  |  |  | Δ | Δ |  |  | Δ |  |  |  | 0.97% |  | Δ | Δ |  | *rbsD*–*[yieO]* | *rbsD, rbsA, rbsC, rbsB, rbsK, rbsR, [yieO]* |
| position | mutation | REL768 Ara+1 500 gen | REL958 Ara+1 1000 gen | REL769 Ara+2 500 gen | REL959 Ara+2 1000 gen | REL770 Ara+3 500 gen | REL960 Ara+3 1000 gen | REL771 Ara+4 500 gen | REL961 Ara+4 1000 gen | REL772 Ara+5 500 gen | REL962 Ara+5 1000 gen | REL773 Ara+6 500 gen | REL963 Ara+6 1000 gen | REL762 Ara−1 500 gen | REL964 Ara−1 1000 gen | REL763 Ara−2 500 gen | REL965 Ara−2 1000 gen | REL764 Ara−3 500 gen | REL966 Ara−3 1000 gen | REL765 Ara−4 500 gen | REL967 Ara−4 1000 gen | REL766 Ara−5 500 gen | REL968 Ara−5 1000 gen | REL767 Ara−6 500 gen | REL969 Ara−6 1000 gen | annotation | gene | description |
| 3,894,997 | Δ6,417 bp |  |  |  |  |  |  |  |  |  |  |  |  | Δ | Δ | 10.0% |  | Δ |  |  |  | Δ |  | Δ | Δ |  | *rbsD*–*[yieO]* | *rbsD, rbsA, rbsC, rbsB, rbsK, rbsR, [yieO]* |
| 3,894,997 | Δ6,414 bp |  |  |  |  |  |  |  | 15.4% |  |  |  |  | Δ | Δ | Δ |  | Δ | 9.8% |  |  | Δ |  | Δ | Δ |  | *rbsD*–*[yieO]* | *rbsD, rbsA, rbsC, rbsB, rbsK, rbsR, [yieO]* |
| 3,894,997 | Δ6,406 bp |  |  |  |  |  | 15.9% |  | Δ |  |  |  |  | Δ | Δ | Δ |  | Δ | 34.3% |  |  | Δ |  | Δ | Δ |  | *rbsD*–*[yieO]* | *rbsD, rbsA, rbsC, rbsB, rbsK, rbsR, [yieO]* |
| 3,894,997 | Δ6,405 bp |  |  |  |  |  | Δ |  | Δ |  |  |  |  | Δ | 6.5% | Δ |  | Δ | Δ |  |  | Δ |  | Δ | Δ |  | *rbsD*–*[yieO]* | *rbsD, rbsA, rbsC, rbsB, rbsK, rbsR, [yieO]* |
| 3,894,997 | Δ6,244 bp | 14.9% |  |  |  |  | Δ |  | Δ |  |  |  |  | Δ | Δ | Δ |  | Δ | Δ |  |  | Δ |  | Δ | Δ |  | *rbsD*–*[yieO]* | *rbsD, rbsA, rbsC, rbsB, rbsK, rbsR, [yieO]* |
| 3,894,997 | Δ6,234 bp | Δ |  |  |  |  | Δ |  | 11.2% |  |  |  |  | Δ | Δ | Δ |  | Δ | Δ |  |  | Δ |  | Δ | Δ |  | *rbsD*–*[yieO]* | *rbsD, rbsA, rbsC, rbsB, rbsK, rbsR, [yieO]* |
| 3,894,997 | Δ6,138 bp | Δ | 5.3% |  |  |  | Δ |  | Δ |  |  |  |  | Δ | Δ | Δ |  | Δ | Δ |  |  | Δ |  | Δ | Δ |  | *rbsD*–*[yieO]* | *rbsD, rbsA, rbsC, rbsB, rbsK, rbsR, [yieO]* |
| 3,894,997 | Δ5,987 bp | Δ | Δ |  |  |  | Δ |  | Δ |  |  |  |  | Δ | Δ | Δ |  | 1.6% | Δ |  |  | Δ |  | Δ | Δ |  | *rbsD*–*[rbsR]* | *rbsD, rbsA, rbsC, rbsB, rbsK, [rbsR]* |
| 3,894,997 | Δ5,627 bp | Δ | Δ |  |  |  | Δ |  | Δ |  |  |  |  | Δ | Δ | Δ |  | Δ | Δ |  |  | 37.8% | 73.6% | Δ | Δ |  | *rbsD*–*[rbsR]* | *rbsD, rbsA, rbsC, rbsB, rbsK, [rbsR]* |
| 3,894,997 | Δ5,458 bp | Δ | Δ |  |  |  | Δ |  | Δ |  |  |  |  | Δ | Δ | Δ |  | Δ | Δ |  | 3.5% | Δ | Δ | Δ | Δ |  | *rbsD*–*[rbsR]* | *rbsD, rbsA, rbsC, rbsB, rbsK, [rbsR]* |
| position | mutation | REL768 Ara+1 500 gen | REL958 Ara+1 1000 gen | REL769 Ara+2 500 gen | REL959 Ara+2 1000 gen | REL770 Ara+3 500 gen | REL960 Ara+3 1000 gen | REL771 Ara+4 500 gen | REL961 Ara+4 1000 gen | REL772 Ara+5 500 gen | REL962 Ara+5 1000 gen | REL773 Ara+6 500 gen | REL963 Ara+6 1000 gen | REL762 Ara−1 500 gen | REL964 Ara−1 1000 gen | REL763 Ara−2 500 gen | REL965 Ara−2 1000 gen | REL764 Ara−3 500 gen | REL966 Ara−3 1000 gen | REL765 Ara−4 500 gen | REL967 Ara−4 1000 gen | REL766 Ara−5 500 gen | REL968 Ara−5 1000 gen | REL767 Ara−6 500 gen | REL969 Ara−6 1000 gen | annotation | gene | description |
| 3,894,997 | Δ5,111 bp | Δ | Δ |  |  |  | Δ |  | Δ |  | 15.6% |  |  | Δ | Δ | Δ |  | Δ | Δ |  | Δ | Δ | Δ | Δ | Δ |  | *rbsD*–*[rbsR]* | *rbsD, rbsA, rbsC, rbsB, rbsK, [rbsR]* |
| 3,894,997 | Δ4,772 bp | 14.2% | Δ |  |  |  | Δ |  | Δ |  | Δ |  |  | Δ | Δ | Δ |  | Δ | Δ |  | Δ | Δ | Δ | Δ | Δ |  | *rbsD*–*[rbsK]* | *rbsD, rbsA, rbsC, rbsB, [rbsK]* |
| 3,894,997 | Δ4,561 bp | Δ | Δ |  |  |  | 29.9% |  | Δ |  | Δ |  |  | Δ | Δ | Δ |  | Δ | Δ |  | Δ | Δ | Δ | Δ | Δ |  | *rbsD*–*[rbsK]* | *rbsD, rbsA, rbsC, rbsB, [rbsK]* |
| 3,894,997 | Δ3,947 bp | Δ | Δ |  |  |  | Δ |  | 44.8% |  | Δ |  |  | Δ | Δ | Δ |  | Δ | Δ |  | Δ | Δ | Δ | Δ | Δ |  | *rbsD*–*[rbsB]* | *rbsD, rbsA, rbsC, [rbsB]* |
| 3,894,997 | Δ3,138 bp | Δ | Δ |  |  |  | Δ |  | Δ |  | Δ |  |  | Δ | Δ | Δ |  | Δ | Δ |  | Δ | Δ | Δ | Δ | 1.8% |  | *rbsD*–*[rbsB]* | *rbsD, rbsA, rbsC, [rbsB]* |
| 3,894,997 | Δ3,064 bp | Δ | Δ |  |  |  | Δ |  | Δ |  | Δ |  |  | Δ | Δ | Δ |  | Δ | Δ |  | Δ | 2.5% | Δ | Δ | Δ |  | *rbsD*–*[rbsC]* | *rbsD, rbsA, [rbsC]* |
| 3,894,997 | Δ2,873 bp | Δ | Δ |  |  |  | Δ |  | Δ |  | Δ |  |  | Δ | Δ | Δ |  | Δ | Δ |  | Δ | 2.4% | Δ | Δ | Δ |  | *rbsD*–*[rbsC]* | *rbsD, rbsA, [rbsC]* |
| 3,894,997 | Δ2,543 bp | 49.6% | 89.1% |  |  |  | Δ |  | Δ |  | Δ |  |  | 0.82% | Δ | Δ |  | Δ | Δ |  | Δ | Δ | Δ | Δ | Δ |  | *rbsD*–*[rbsC]* | *rbsD, rbsA, [rbsC]* |
| 3,894,997 | Δ2,296 bp | Δ | Δ |  |  |  | Δ |  | Δ |  | Δ |  |  | Δ | Δ | Δ |  | Δ | 8.6% |  | Δ | Δ | Δ | Δ | Δ |  | *rbsD*–*[rbsC]* | *rbsD, rbsA, [rbsC]* |
| 3,894,997 | Δ1,799 bp | Δ | Δ |  |  |  | Δ |  | Δ | 0.93% | 19.7% |  |  | Δ | Δ | Δ |  | Δ | Δ |  | Δ | Δ | Δ | Δ | Δ |  | *rbsD*–*[rbsA]* | *rbsD, [rbsA]* |
| position | mutation | REL768 Ara+1 500 gen | REL958 Ara+1 1000 gen | REL769 Ara+2 500 gen | REL959 Ara+2 1000 gen | REL770 Ara+3 500 gen | REL960 Ara+3 1000 gen | REL771 Ara+4 500 gen | REL961 Ara+4 1000 gen | REL772 Ara+5 500 gen | REL962 Ara+5 1000 gen | REL773 Ara+6 500 gen | REL963 Ara+6 1000 gen | REL762 Ara−1 500 gen | REL964 Ara−1 1000 gen | REL763 Ara−2 500 gen | REL965 Ara−2 1000 gen | REL764 Ara−3 500 gen | REL966 Ara−3 1000 gen | REL765 Ara−4 500 gen | REL967 Ara−4 1000 gen | REL766 Ara−5 500 gen | REL968 Ara−5 1000 gen | REL767 Ara−6 500 gen | REL969 Ara−6 1000 gen | annotation | gene | description |
| 3,894,997 | Δ1,713 bp | Δ | Δ |  |  |  | Δ |  | 9.5% | Δ | Δ |  |  | Δ | Δ | Δ |  | Δ | Δ |  | Δ | Δ | Δ | Δ | Δ |  | *rbsD*–*[rbsA]* | *rbsD, [rbsA]* |
| 3,894,997 | Δ1,160 bp | Δ | Δ |  |  |  | Δ |  | Δ | Δ | Δ |  |  | Δ | Δ | Δ |  | Δ | Δ |  | 6.5% | Δ | Δ | Δ | Δ |  | *rbsD*–*[rbsA]* | *rbsD, [rbsA]* |
| 3,894,997 | Δ1,138 bp | Δ | Δ |  |  |  | Δ |  | Δ | Δ | Δ |  |  | 4.0% | Δ | Δ |  | Δ | Δ |  | Δ | Δ | Δ | Δ | Δ |  | *rbsD*–*[rbsA]* | *rbsD, [rbsA]* |
| 3,894,997 | Δ774 bp | Δ | Δ |  |  |  | Δ |  | Δ | Δ | Δ |  |  | Δ | Δ | Δ |  | Δ | Δ |  | Δ | 3.1% | Δ | Δ | Δ |  | *rbsD*–*[rbsA]* | *rbsD, [rbsA]* |
| 3,894,997 | Δ730 bp | 4.8% | Δ |  |  |  | Δ |  | Δ | Δ | Δ |  |  | Δ | Δ | Δ |  | Δ | Δ |  | Δ | Δ | Δ | Δ | Δ |  | *rbsD*–*[rbsA]* | *rbsD, [rbsA]* |
| 3,894,997 | Δ391 bp | Δ | Δ |  |  |  | Δ |  | Δ | Δ | Δ |  |  | Δ | Δ | Δ |  | Δ | Δ |  | Δ | 3.8% | Δ | Δ | Δ |  | *[rbsD]* | *[rbsD]* |
| 3,894,997 | Δ283 bp | Δ | Δ |  |  |  | Δ |  | Δ | Δ | 12.1% |  |  | Δ | Δ | Δ |  | Δ | Δ |  | Δ | Δ | Δ | Δ | Δ |  | *[rbsD]* | *[rbsD]* |
| 3,894,998 | Δ5,122 bp | Δ | Δ |  |  |  | Δ |  | Δ |  |  |  |  | Δ | Δ | Δ |  | Δ | Δ |  | 5.3% | Δ | Δ | Δ | Δ |  | *rbsD*–*[rbsR]* | *rbsD, rbsA, rbsC, rbsB, rbsK, [rbsR]* |
| 3,894,998 | Δ4,604 bp | Δ | Δ |  |  |  | Δ |  | Δ |  | Δ |  |  | Δ | Δ | Δ |  | Δ | Δ |  | Δ | Δ | Δ | 1.7% | Δ |  | *rbsD*–*[rbsK]* | *rbsD, rbsA, rbsC, rbsB, [rbsK]* |
| 3,894,998 | Δ3,310 bp | Δ | Δ |  |  |  | Δ |  | Δ |  | Δ |  |  | Δ | Δ | Δ |  | Δ | Δ |  | Δ | 1.8% | Δ | Δ | Δ |  | *rbsD*–*[rbsB]* | *rbsD, rbsA, rbsC, [rbsB]* |
| position | mutation | REL768 Ara+1 500 gen | REL958 Ara+1 1000 gen | REL769 Ara+2 500 gen | REL959 Ara+2 1000 gen | REL770 Ara+3 500 gen | REL960 Ara+3 1000 gen | REL771 Ara+4 500 gen | REL961 Ara+4 1000 gen | REL772 Ara+5 500 gen | REL962 Ara+5 1000 gen | REL773 Ara+6 500 gen | REL963 Ara+6 1000 gen | REL762 Ara−1 500 gen | REL964 Ara−1 1000 gen | REL763 Ara−2 500 gen | REL965 Ara−2 1000 gen | REL764 Ara−3 500 gen | REL966 Ara−3 1000 gen | REL765 Ara−4 500 gen | REL967 Ara−4 1000 gen | REL766 Ara−5 500 gen | REL968 Ara−5 1000 gen | REL767 Ara−6 500 gen | REL969 Ara−6 1000 gen | annotation | gene | description |
| 3,894,998 | Δ3,060 bp | Δ | Δ |  |  |  | Δ |  | Δ |  | Δ |  |  | 29.3% | 48.9% | Δ |  | Δ | Δ |  | Δ | Δ | Δ | Δ | Δ |  | *rbsD*–*[rbsC]* | *rbsD, rbsA, [rbsC]* |
| 3,894,998 | Δ613 bp | Δ | Δ |  |  |  | Δ |  | Δ | 8.1% | 21.5% |  |  | Δ | Δ | Δ |  | Δ | Δ |  | Δ | Δ | Δ | Δ | Δ |  | *rbsD*–*[rbsA]* | *rbsD, [rbsA]* |
| 3,894,999 | Δ7,233 bp | 2.1% |  |  |  |  |  |  |  |  |  |  |  |  |  |  |  | Δ |  |  |  |  |  | Δ |  |  | *rbsD*–*[yieO]* | *rbsD, rbsA, rbsC, rbsB, rbsK, rbsR, [yieO]* |
| 3,894,999 | Δ5,691 bp | Δ | Δ |  |  |  | 9.3% |  | Δ |  |  |  |  | Δ | Δ | Δ |  | Δ | Δ |  |  | Δ |  | Δ | Δ |  | *rbsD*–*[rbsR]* | *rbsD, rbsA, rbsC, rbsB, rbsK, [rbsR]* |
| 3,894,999 | Δ1,317 bp | Δ | Δ |  |  | 7.9% | Δ |  | Δ | Δ | Δ |  |  | Δ | Δ | Δ |  | Δ | Δ |  | Δ | Δ | Δ | Δ | Δ |  | *rbsD*–*[rbsA]* | *rbsD, [rbsA]* |
| 3,895,000 | Δ7,170 bp | Δ |  |  |  |  |  |  |  |  |  |  |  |  |  |  |  | Δ |  |  | 9.4% |  |  | Δ |  |  | *rbsD*–*[yieO]* | *rbsD, rbsA, rbsC, rbsB, rbsK, rbsR, [yieO]* |
| 4,011,948 | T→A |  |  |  |  |  |  |  |  | 25.9% | 24.5% |  |  |  |  |  |  |  |  |  |  |  |  |  |  | V144E (GTG→GAG) | *trkH* → | potassium transporter |
| 4,012,725 | C→T |  |  |  |  |  |  |  |  | 39.8% | 23.3% |  |  |  |  |  |  |  |  |  |  |  |  |  |  | A403V (GCC→GTC) | *trkH* → | potassium transporter |
| 4,072,147 | G→T |  |  |  |  |  |  |  |  |  |  |  |  |  |  |  |  |  | 8.7% |  |  |  |  |  |  | R234L (CGC→CTC) | *ECB\_03786* → | putative glycoporin |
| 4,100,192 | G→A |  |  |  |  |  |  |  |  |  |  |  |  |  | 8.4% |  |  |  |  |  |  |  |  |  |  | P347S (CCG→TCG) | *hslU* ← | ATP‑dependent protease ATP‑binding subunit |
| position | mutation | REL768 Ara+1 500 gen | REL958 Ara+1 1000 gen | REL769 Ara+2 500 gen | REL959 Ara+2 1000 gen | REL770 Ara+3 500 gen | REL960 Ara+3 1000 gen | REL771 Ara+4 500 gen | REL961 Ara+4 1000 gen | REL772 Ara+5 500 gen | REL962 Ara+5 1000 gen | REL773 Ara+6 500 gen | REL963 Ara+6 1000 gen | REL762 Ara−1 500 gen | REL964 Ara−1 1000 gen | REL763 Ara−2 500 gen | REL965 Ara−2 1000 gen | REL764 Ara−3 500 gen | REL966 Ara−3 1000 gen | REL765 Ara−4 500 gen | REL967 Ara−4 1000 gen | REL766 Ara−5 500 gen | REL968 Ara−5 1000 gen | REL767 Ara−6 500 gen | REL969 Ara−6 1000 gen | annotation | gene | description |
| 4,100,992 | T→G |  |  |  |  |  | 12.3% |  |  |  |  |  |  |  |  |  |  |  |  |  |  |  |  |  |  | K80T (AAA→ACA) | *hslU* ← | ATP‑dependent protease ATP‑binding subunit |
| 4,127,995 | G→T |  |  |  |  |  |  |  |  |  |  |  |  |  | 32.0% |  |  |  |  |  |  |  |  |  |  | L144I (CTT→ATT) | *yijO* ← | predicted DNA‑binding transcriptional regulator |
| 4,141,016 | C→A |  |  |  |  |  |  |  |  |  |  |  |  | 42.9% | 64.9% | 10.0% |  |  |  |  |  |  |  |  |  | T30N (ACC→AAC) | *yijC* → | DNA‑binding transcriptional repressor |
| 4,141,099 | C→T |  |  |  |  |  |  |  |  | 15.0% | 34.9% |  |  |  |  |  |  |  |  |  |  |  |  |  |  | R58C (CGT→TGT) | *yijC* → | DNA‑binding transcriptional repressor |
| 4,141,135 | C→T |  | 8.0% |  |  |  |  |  |  |  |  |  |  |  |  |  |  |  |  |  |  |  |  |  |  | H70Y (CAT→TAT) | *yijC* → | DNA‑binding transcriptional repressor |
| 4,141,180 | A→C |  | 28.2% |  |  |  |  |  |  |  |  |  |  |  |  |  |  |  |  |  |  |  |  |  |  | S85R (AGC→CGC) | *yijC* → | DNA‑binding transcriptional repressor |
| 4,141,192 | C→A |  |  |  |  |  |  |  |  |  |  |  |  |  |  |  |  |  |  |  |  | 5.7% |  |  |  | L89I (CTA→ATA) | *yijC* → | DNA‑binding transcriptional repressor |
| 4,141,441 | C→T |  |  |  |  |  |  |  |  |  |  |  |  |  |  |  |  |  | 100% |  |  |  |  |  |  | Q172\* (CAA→TAA) | *yijC* → | DNA‑binding transcriptional repressor |
| 4,141,545 | IS*150* (+) +3 bp |  |  |  |  |  |  |  |  |  |  |  |  |  |  |  |  | 1.8% |  |  |  |  |  |  |  | coding (618‑620/705 nt) | *yijC* → | DNA‑binding transcriptional repressor |
| 4,141,561 | G→A |  |  |  |  |  |  |  |  |  |  |  |  |  |  |  |  |  |  |  |  |  |  | 39.3% |  | G212R (GGG→AGG) | *yijC* → | DNA‑binding transcriptional repressor |
| position | mutation | REL768 Ara+1 500 gen | REL958 Ara+1 1000 gen | REL769 Ara+2 500 gen | REL959 Ara+2 1000 gen | REL770 Ara+3 500 gen | REL960 Ara+3 1000 gen | REL771 Ara+4 500 gen | REL961 Ara+4 1000 gen | REL772 Ara+5 500 gen | REL962 Ara+5 1000 gen | REL773 Ara+6 500 gen | REL963 Ara+6 1000 gen | REL762 Ara−1 500 gen | REL964 Ara−1 1000 gen | REL763 Ara−2 500 gen | REL965 Ara−2 1000 gen | REL764 Ara−3 500 gen | REL966 Ara−3 1000 gen | REL765 Ara−4 500 gen | REL967 Ara−4 1000 gen | REL766 Ara−5 500 gen | REL968 Ara−5 1000 gen | REL767 Ara−6 500 gen | REL969 Ara−6 1000 gen | annotation | gene | description |
| 4,163,697 | (AGACAAACGTGCGCTGGAAA)1→2 |  |  |  |  |  |  |  |  | 0.94% |  |  |  |  |  |  |  |  |  |  |  |  |  |  |  | coding (2842/4029 nt) | *rpoB* → | DNA‑directed RNA polymerase subunit beta |
| 4,164,259 | A→G |  |  |  |  |  |  |  | 5.5% |  |  |  |  |  |  |  |  |  |  |  |  |  |  |  |  | Q1135R (CAG→CGG) | *rpoB* → | DNA‑directed RNA polymerase subunit beta |
| 4,181,329 | T→A |  |  |  |  |  |  |  |  |  | 16.6% |  |  |  |  |  |  |  |  |  |  |  |  |  |  | intergenic (‑35/‑203) | *zraP* ← / → *zraS* | Zn‑binding periplasmic protein/sensory histidine kinase in two‑component regulatory system with ZraR |
| 4,202,333 | A→G |  |  |  |  |  |  |  |  |  |  |  |  |  |  |  |  |  |  |  | 5.5% |  |  |  |  | V76A (GTC→GCC) | *iclR* ← | DNA‑binding transcriptional repressor |
| 4,212,323 | IS*186* (–) |  |  |  |  |  |  |  |  |  |  |  |  |  |  |  |  | 4.8% |  |  |  |  |  |  |  | intergenic (‑159/‑367) | *lysC* ← / → *pgi* | aspartate kinase III/glucose‑6‑phosphate isomerase |
| 4,412,814 | G→A |  | 7.1% |  |  |  |  |  |  |  |  |  |  |  |  |  |  |  |  |  |  |  |  |  |  | Q16Q (CAG→CAA) | *ytfA* → | predicted transcriptional regulator |
| 4,431,394 | Δ1 bp |  |  |  |  |  |  |  |  |  |  |  |  |  |  |  |  | 22.7% |  |  |  |  |  |  |  | coding (3526/3780 nt) | *ytfN* → | hypothetical protein |
| 4,431,395 | Δ1 bp |  |  |  |  |  |  |  |  |  |  |  |  |  |  |  |  | 23.0% |  |  |  |  |  |  |  | coding (3527/3780 nt) | *ytfN* → | hypothetical protein |
| 4,518,768 | Δ1 bp |  |  |  |  |  |  |  |  |  |  |  |  |  |  |  |  |  | 5.2% |  |  |  |  |  |  | coding (136/1107 nt) | *yjhT* ← | hypothetical protein |
| 4,530,778 | (AACCCT)1→2 |  |  |  |  |  |  |  |  |  |  |  |  |  |  |  |  | 30.4% | 92.4% |  |  |  |  |  |  | intergenic (+154/+89) | *fimH* → / ← *gntP* | minor component of type 1 fimbriae/fructuronate transporter |
| position | mutation | REL768 Ara+1 500 gen | REL958 Ara+1 1000 gen | REL769 Ara+2 500 gen | REL959 Ara+2 1000 gen | REL770 Ara+3 500 gen | REL960 Ara+3 1000 gen | REL771 Ara+4 500 gen | REL961 Ara+4 1000 gen | REL772 Ara+5 500 gen | REL962 Ara+5 1000 gen | REL773 Ara+6 500 gen | REL963 Ara+6 1000 gen | REL762 Ara−1 500 gen | REL964 Ara−1 1000 gen | REL763 Ara−2 500 gen | REL965 Ara−2 1000 gen | REL764 Ara−3 500 gen | REL966 Ara−3 1000 gen | REL765 Ara−4 500 gen | REL967 Ara−4 1000 gen | REL766 Ara−5 500 gen | REL968 Ara−5 1000 gen | REL767 Ara−6 500 gen | REL969 Ara−6 1000 gen | annotation | gene | description |
| 4,558,578 | Δ1 bp |  |  |  |  |  |  |  | 6.2% |  |  |  |  |  |  |  |  |  |  |  |  |  |  |  |  | coding (860/1425 nt) | *hsdS* ← | specificity determinant for hsdM and hsdR |
| 4,615,674 | IS*150* (+) +3 bp |  |  |  |  |  |  |  |  |  |  |  |  |  |  |  |  |  |  |  |  |  | 20.2% |  |  | coding (146‑148/1233 nt) | *nadR* → | nicotinamide‑nucleotide adenylyltransferase |
| 4,615,842 | A→T |  |  |  |  |  |  |  |  |  |  |  |  |  |  |  |  |  |  |  |  |  |  |  | 87.8% | D105V (GAT→GTT) | *nadR* → | nicotinamide‑nucleotide adenylyltransferase |
| 4,616,212 | Δ1 bp | 43.3% | 86.3% |  |  |  |  |  |  |  |  |  |  |  |  |  |  |  |  |  |  |  |  |  |  | coding (684/1233 nt) | *nadR* → | nicotinamide‑nucleotide adenylyltransferase |
| 4,616,296 | T→G |  |  |  |  |  |  |  |  |  | 14.4% |  |  |  |  |  |  |  |  |  |  |  |  |  |  | N256K (AAT→AAG) | *nadR* → | nicotinamide‑nucleotide adenylyltransferase |
